# Supplementary figures and images for: ATG9A regulates the dissociation of recycling endosomes from microtubules to form liquid influenza A virus inclusions
Source: PLoS Biol. 2023 Nov 20;21(11):e3002290. doi: 10.1371/journal.pbio.3002290 (PMC10695400; doi:10.1371/journal.pbio.3002290)

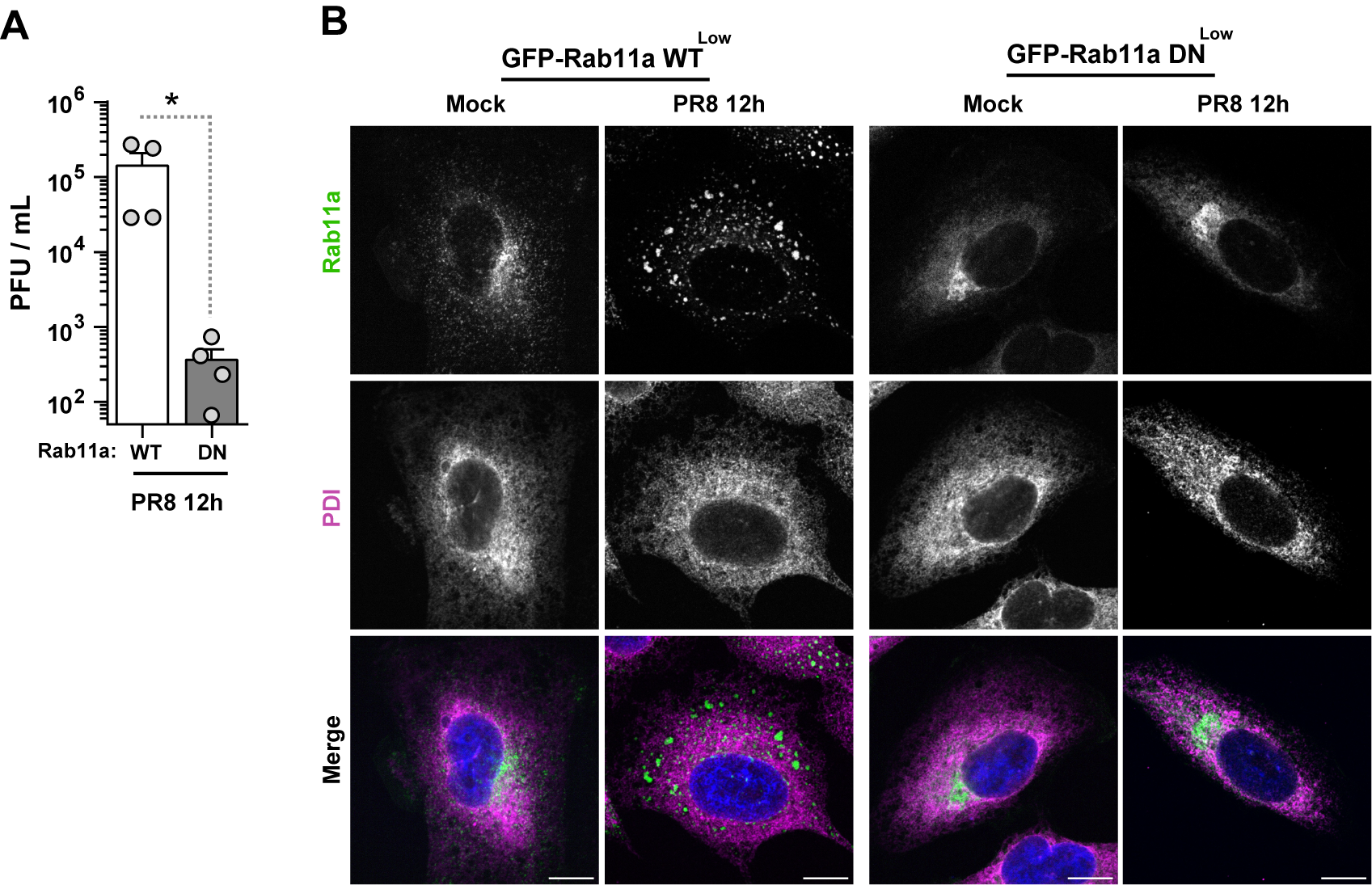

Supplement: S1 Fig — (A) Cells (GFP-Rab11a WTlow and GFP-Rab11a DNlow) were infected or mock-infected with PR8 virus for 12 h, at an MOI of 3. Viral production was determined at 12 h postinfection by plaque assay and plotted as PFUs per milliliter (mL) SEM. Data are a pool from 4 independent experiments. Statistical analysis was done by Mann–Whitney test (*p < 0.05). (B) Formation of viral inclusions was analyzed by immunofluorescence at 12 h postinfection, using GFP-Rab11a as a proxy to detect viral inclusions. The ER distribution was detected by antibody staining against host PDI (gray). Infected cells expressing Rab11a WT were able to mount viral inclusions, whereas infected cells expressing Rab11a DN were not. Cells were stained for ER (magenta) as a cellular reference. Bar = 10 μm. ER, endoplasmic reticulum MOI, multiplicity of infection; PFU, plaque-forming unit; SEM, standard error of the mean; WT, wild-type. (TIF) [file pbio.3002290.s001.tif]

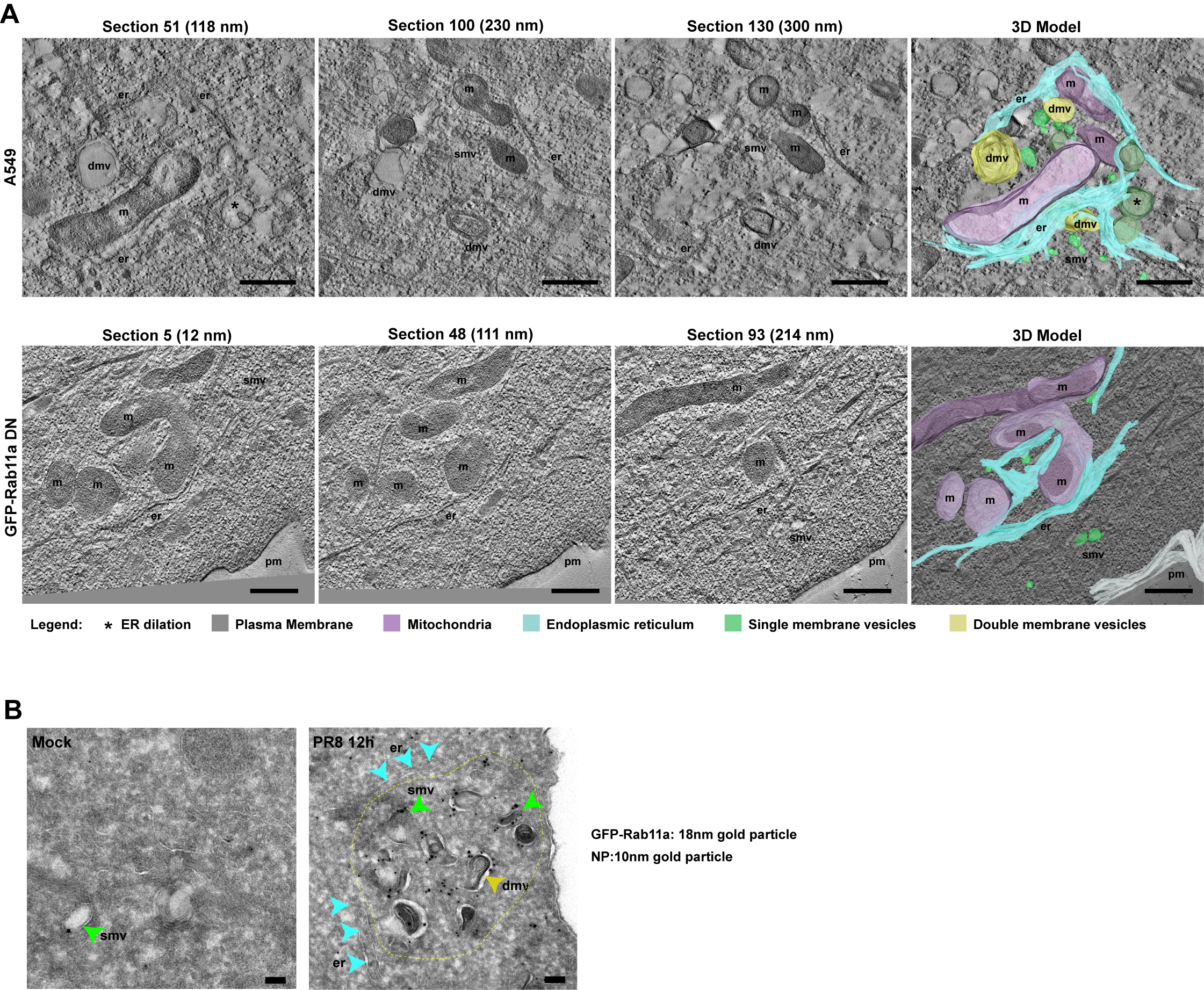

Supplement: S2 Fig — (A) Cells (A549 and GFP-Rab11a DNlow) were infected with PR8 virus for 12 h, at an MOI of 3. Cells were processed by high-pressure freezing/freeze substitution and imaged by ET-TEM. In each case, 4 sequential tomograms (of 120 nm each approximately) were acquired and stitched together. Representative cells are shown with individual sections (including section height in nm) and the 3D cumulative model. For each condition, at least 10 cells were analyzed. Bar = 500 nm. Images were extracted from S7, S8, S9, and S10 Videos. Abbreviations: pm, plasma membrane; er, endoplasmic reticulum; v, budding virions; m, mitochondria; smv, single-membrane vesicle; dmv, double-membrane vesicle. (B) Cells (GFP-Rab11a WTlow) were infected or mock-infected with PR8 virus for 12 h at an MOI of 3. Sections (70 nm) were stained by Tokuyasu double immunogold labeling using antibodies against GFP (18 nm-gold particle to detect Rab11a) and viral NP protein (10 nm-gold particle to detect vRNPs) and imaged by TEM. Green arrowheads show single-membrane vesicles (smv), yellow arrowheads highlight double-membrane vesicles (dmv), and blue arrowheads point to the ER (er). The yellow dashed line delimits a viral inclusion. Bar = 100 nm. ER, endoplasmic reticulum; ET, electron tomography; IAV, influenza A virus; MOI, multiplicity of infection; NP, nucleoprotein; TEM, transmission electron microscopy; vRNP, viral ribonucleoprotein; 3D, 3-dimensional. (TIF) [file pbio.3002290.s002.tif]

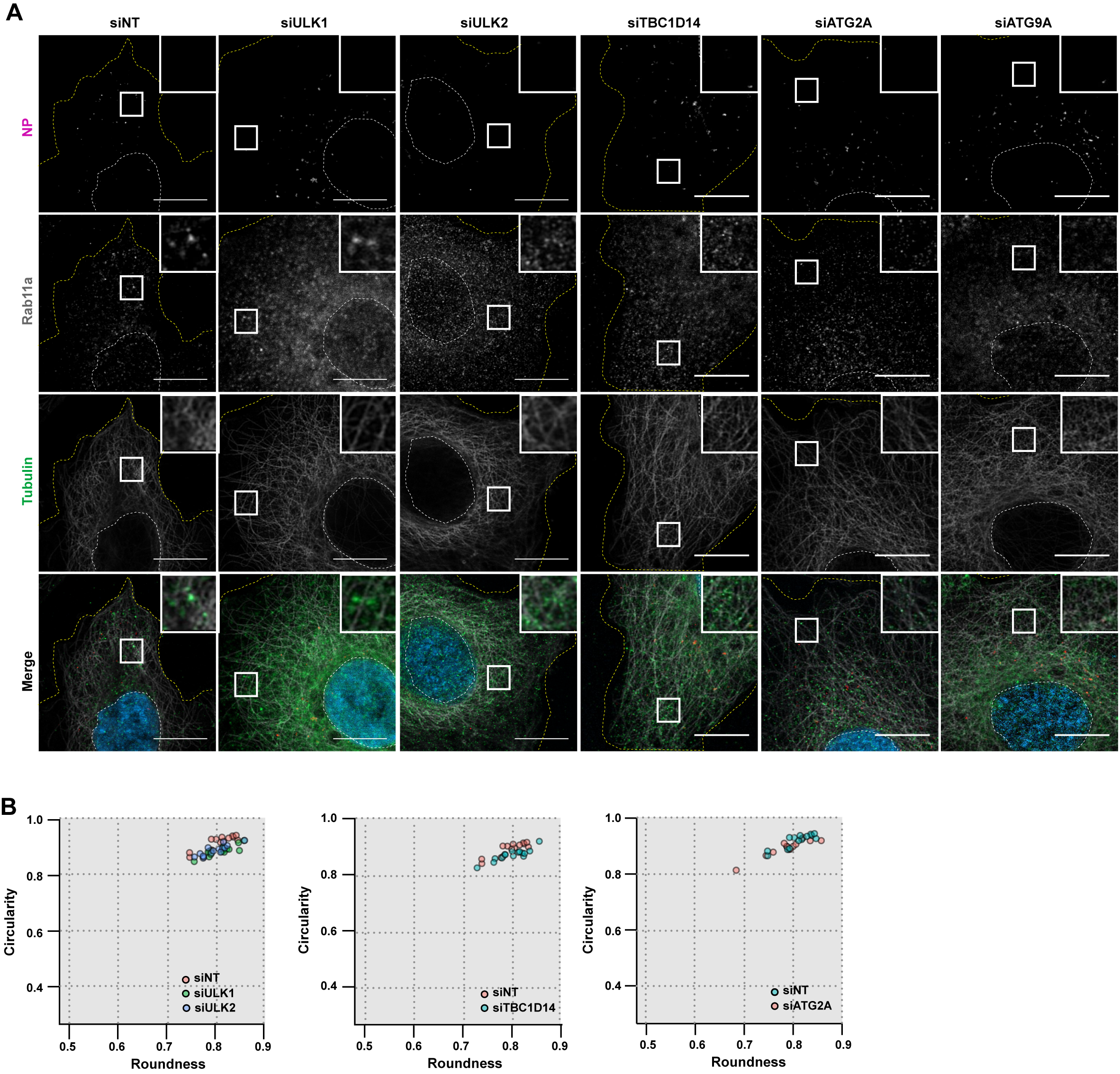

Supplement: S3 Fig — Cells (A549) were treated with siRNA non-targeting (siNT) or targeting ULK1 (siULK1), ULK2 (siULK2), TBC1D14 (siTBC1D14), ATG2A (siATG2A), and ATG9A (siATG9A) for 48 h and then mock-infected or infected with PR8 virus, at an MOI of 3, for 8 h. (A) Mock-infected cells were fixed and analyzed by immunofluorescence using an antibody against viral NP protein (magenta), host Rab11 (gray), and tubulin (green). Representative infected cells are shown in the main text in Fig 3D. (B) In PR8 infected cells, the correlation between roundness and circularity of viral inclusions, as marked by viral NP protein, were calculated for each condition using the Shape Descriptor tool (Image J, NIH) and were plotted as the percentage of a binned frequency distribution as shown. The maximum value of roundness and circularity (1) corresponds to a circular structure, whereas the minimum value represents a linear structure (0). Approximately 15 cells, from 2 independent experiments, were analyzed per condition. Statistical analysis was done by two-way ANOVA, followed by a Sidak’s multiple comparisons test (no statistical differences were found). Circularity and roundness values for the ATG9A depleted condition are shown in the main text in Fig 3F. (TIF) [file pbio.3002290.s003.tif]

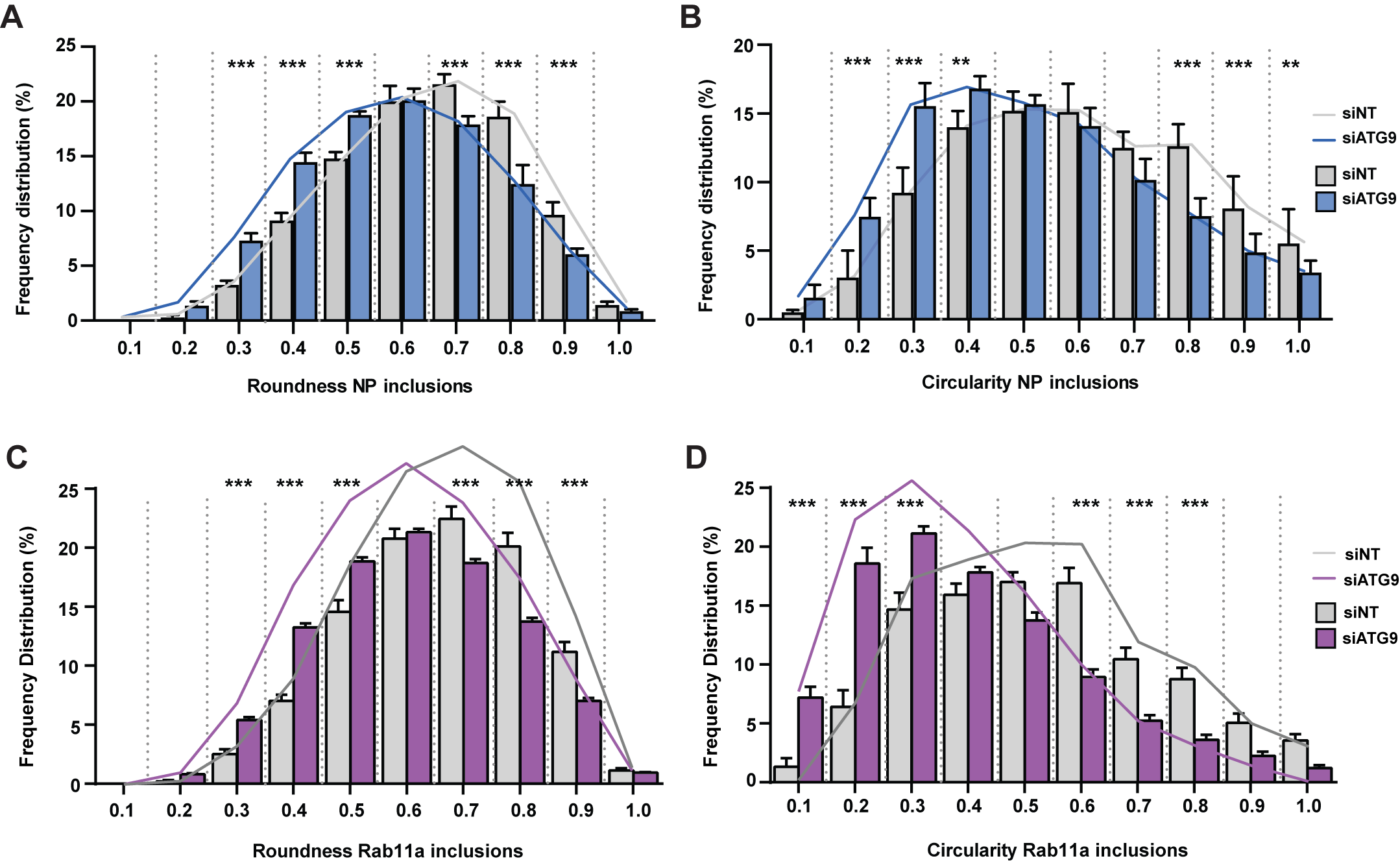

Supplement: S4 Fig — Cells (A549) were treated with siRNA non-targeting (siNT) or targeting ATG9A (siATG9A) for 48 h and then infected, at an MOI of 3, with PR8 virus for 8 h. Cells were fixed and analyzed by immunofluorescence using an antibody against viral NP protein (A, B) or host Rab11 (C, D). The (A, C) roundness and (B, D) circularity of viral inclusions were calculated for each condition using the Shape Descriptor tool (Image J, NIH) and were plotted as the percentage of a binned frequency distribution as shown. The maximum value of roundness and circularity (1) corresponds to a circular structure, whereas the minimum value represents a linear structure (0). More than 80 cells, from 3 independent experiments, were analyzed per condition. Statistical analysis was done by two-way ANOVA, followed by a Sidak’s multiple comparisons test (***p < 0.001, **p < 0.01). (TIF) [file pbio.3002290.s004.tif]

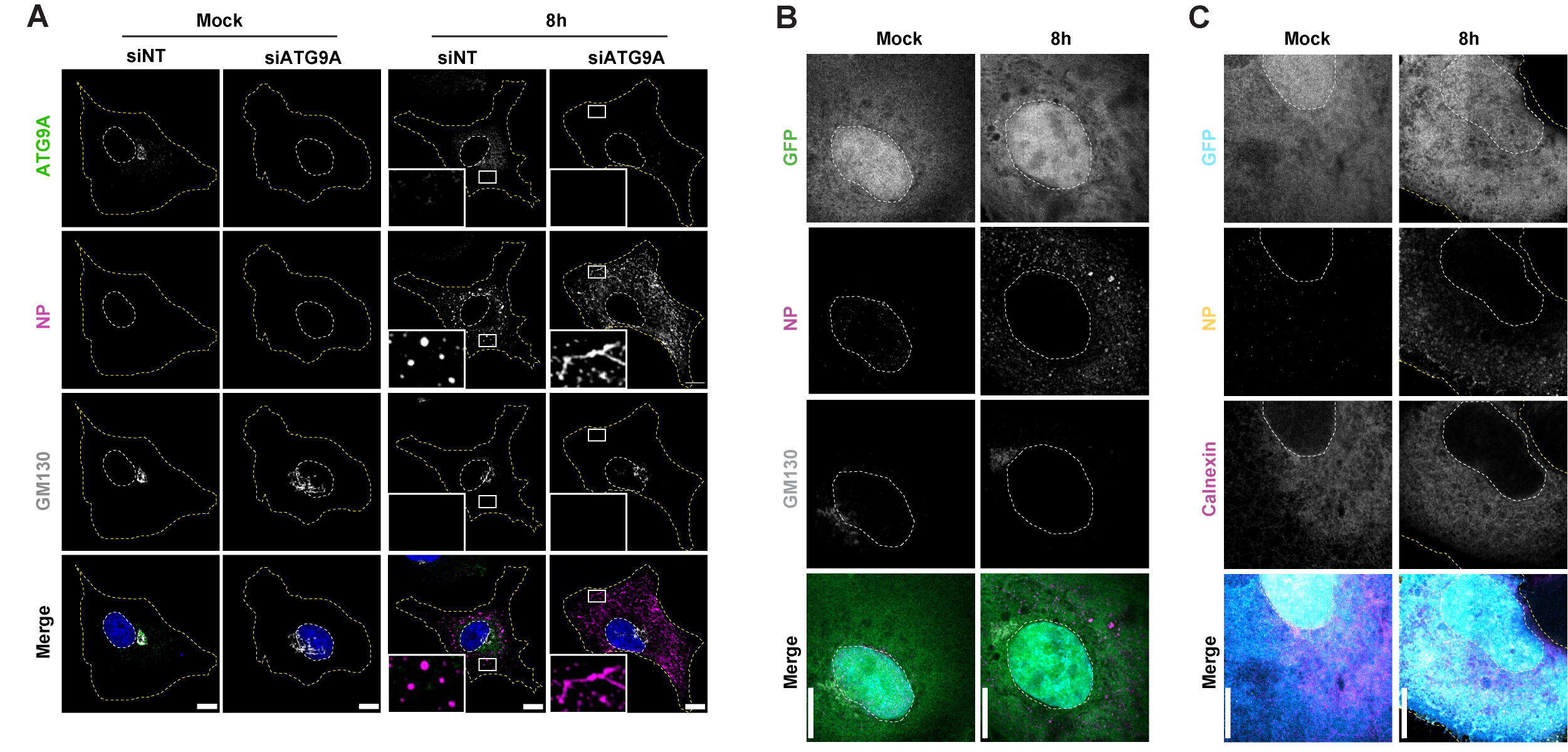

Supplement: S5 Fig — (A) Cells (A549) were treated with siRNA non-targeting (siNT) or targeting ATG9A (siATG9A) for 48 h and then mock-infected or infected, at an MOI of 3, with PR8 virus for 8 h. Cells were fixed and analyzed by immunofluorescence using an antibody against ATG9A (green), viral NP protein (magenta), or host GM130 (gray). Viral inclusions/vRNPs are highlighted by white boxes. Cell periphery and nuclei (blue, Hoechst staining) are delineated by yellow and white dashed lines, respectively. Bar = 10 μm. (B, C) Cells (A549) were transfected with a plasmid encoding GFP (as control for GFP-ATG9A) for 24 h and then infected or mock-infected with PR8 virus, at an MOI of 10, for 8 h. The localization of endogenous host proteins (GM130—Golgi or Calnexin—ER) and viral protein NP was determined by immunofluorescence using antibodies against these proteins. Nuclei (blue or gray, Hoechst staining) and cell periphery are delimited by white and yellow dashed lines, respectively. Bar = 10 μm. ER, endoplasmic reticulum; GFP, green fluorescent protein; IAV, influenza A virus; MOI, multiplicity of infection; NP, nucleoprotein; vRNP, viral ribonucleoprotein. (TIF) [file pbio.3002290.s005.tif]

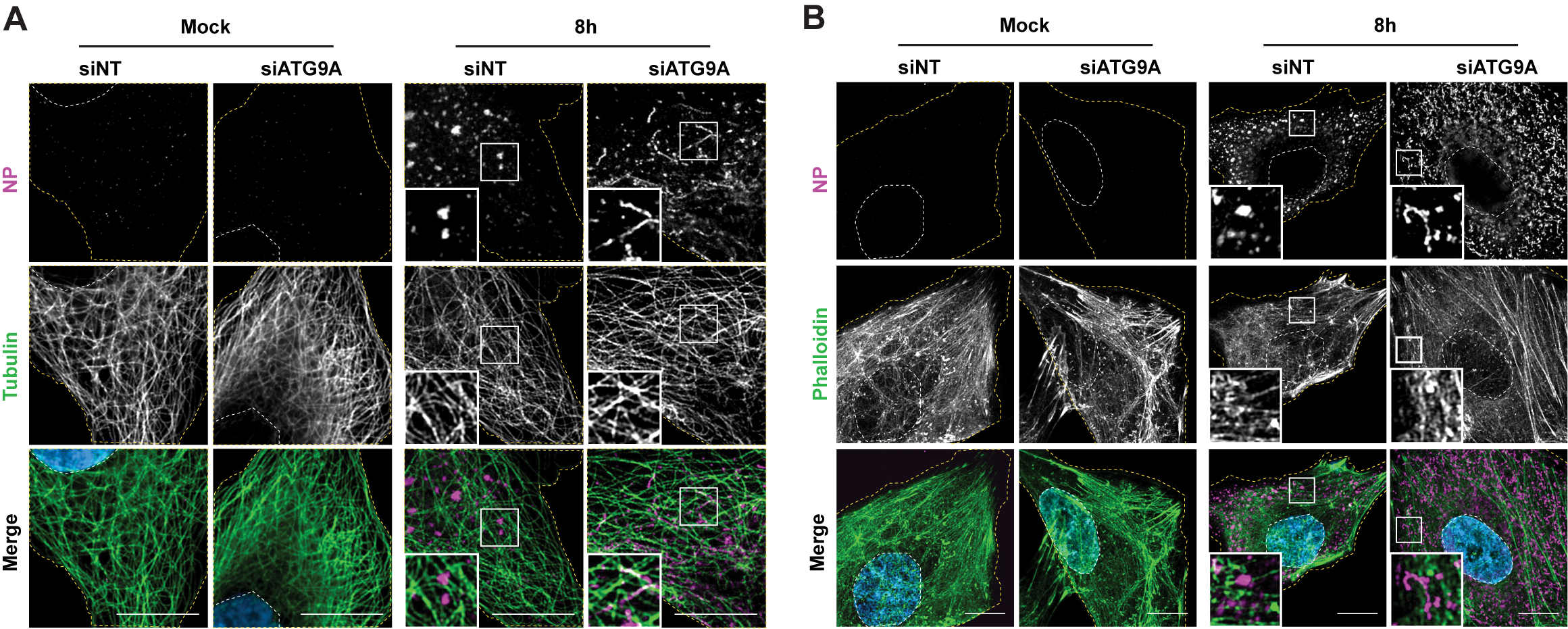

Supplement: S6 Fig — (A, B) Cells (A549) were treated with siRNA non-targeting (siNT) or targeting ATG9A (siATG9A) for 48 h and then infected, at an MOI of 3, with PR8 virus for 8 h. The localization of tubulin (green) and viral protein NP (magenta) was determined by immunofluorescence using antibodies against these proteins. The distribution of actin filaments was determined by staining with the dye phalloidin Alexa Fluor 488 (green). Nuclei (blue, Hoechst staining) and cell periphery are delimited by white and yellow dashed lines, respectively. Viral inclusions are highlighted in white boxes. Bar = 10 μm. (TIF) [file pbio.3002290.s006.tif]

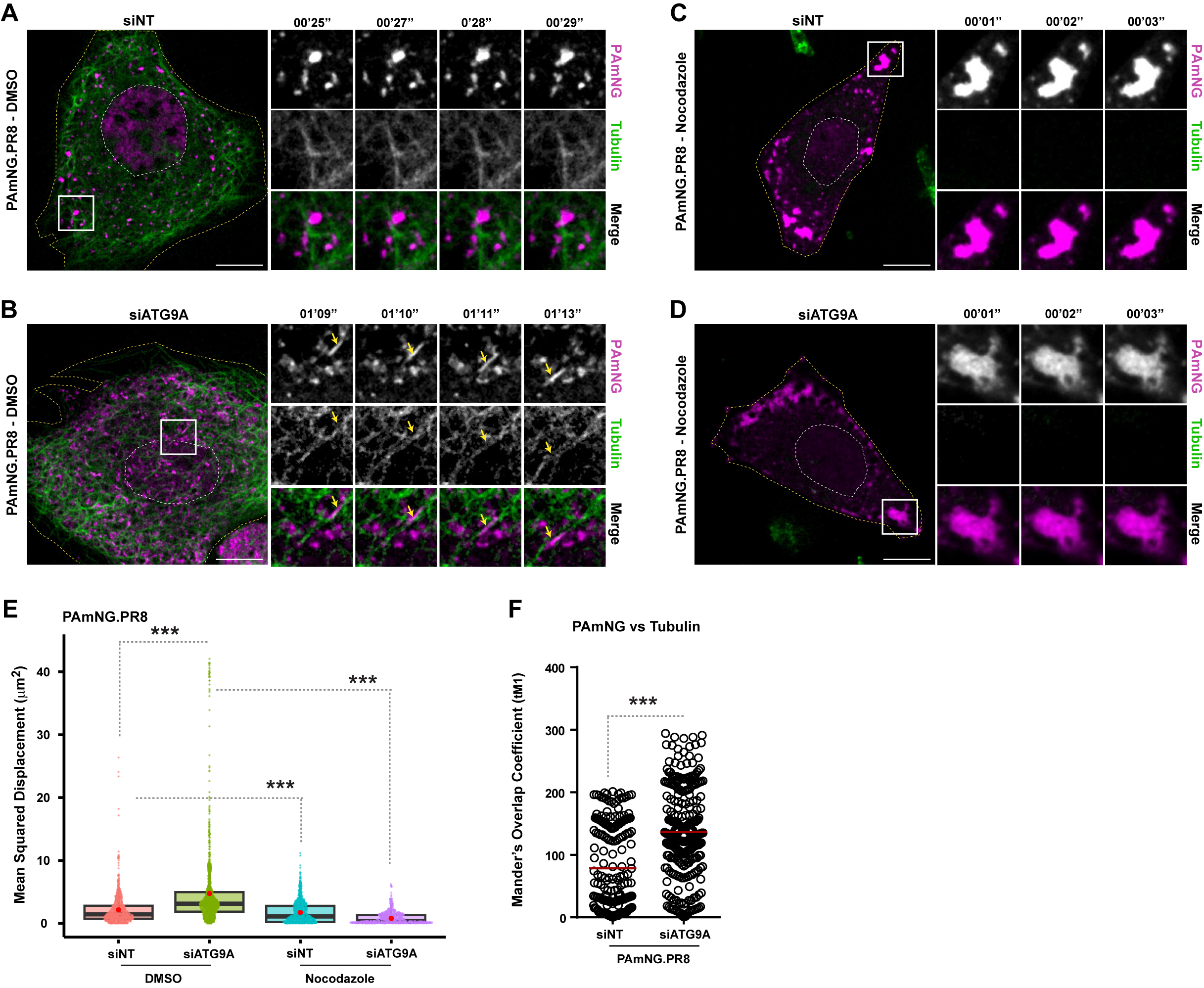

Supplement: S7 Fig — (A, B) Cells (A549) were treated with siRNA non-targeting (siNT) or targeting ATG9A (siATG9A) for 48 h. Upon this period, cells were infected or mock-infected with PA-mNeonGreen PR8 virus (PAmNG.PR8, magenta) for 8 h, at an MOI of 10, and simultaneously treated with 200 nM Sir-Tubulin dye to stain the microtubules (green) in live cells. Cells were imaged for 2 min (2 s/frame) under time-lapse conditions at 8 h postinfection. White boxes show viral inclusions/PA-mNeonGreen. Individual frames with single moving particles highlighted with yellow arrows are shown in the small panels. Bar = 10 μm. Images from selected infected cells were extracted from S15 and S16 Videos. (C, D) Cells (A549) were treated as explained above (in A, B). At 8 h postinfection, cells were treated with DMSO or 10 μg/mL of nocodazole for 2 h. Cells were imaged at 10 h postinfection. White boxes show viral inclusions/PA-mNeonGreen. Bar = 10 μm. Images from selected infected cells were extracted from S17 and S18 Videos. (E) Each viral inclusion in a cell was tracked using the TrackMate plugin (FIJI, NIH) and displacement was quantified as explained in Fig 6G. Data were plotted as the mean squared displacement (MSD, μm2) per treatment. The red dot indicates the median in the boxplots. Statistical analysis was done by a Kruskal–Wallis test (***p < 0.001). (F) Colocalization between microtubules (tubulin) and viral inclusions (PA-mNeonGreen) was determined as the Manders’ Overlap Coefficient tM1 (thresholded; explained in Methods section). Between 9 and 13 cells per condition were analyzed. Statistical analysis was done by a Student t test (***p < 0.001). Experiments were performed twice. (TIF) [file pbio.3002290.s007.tif]

Fig 3C

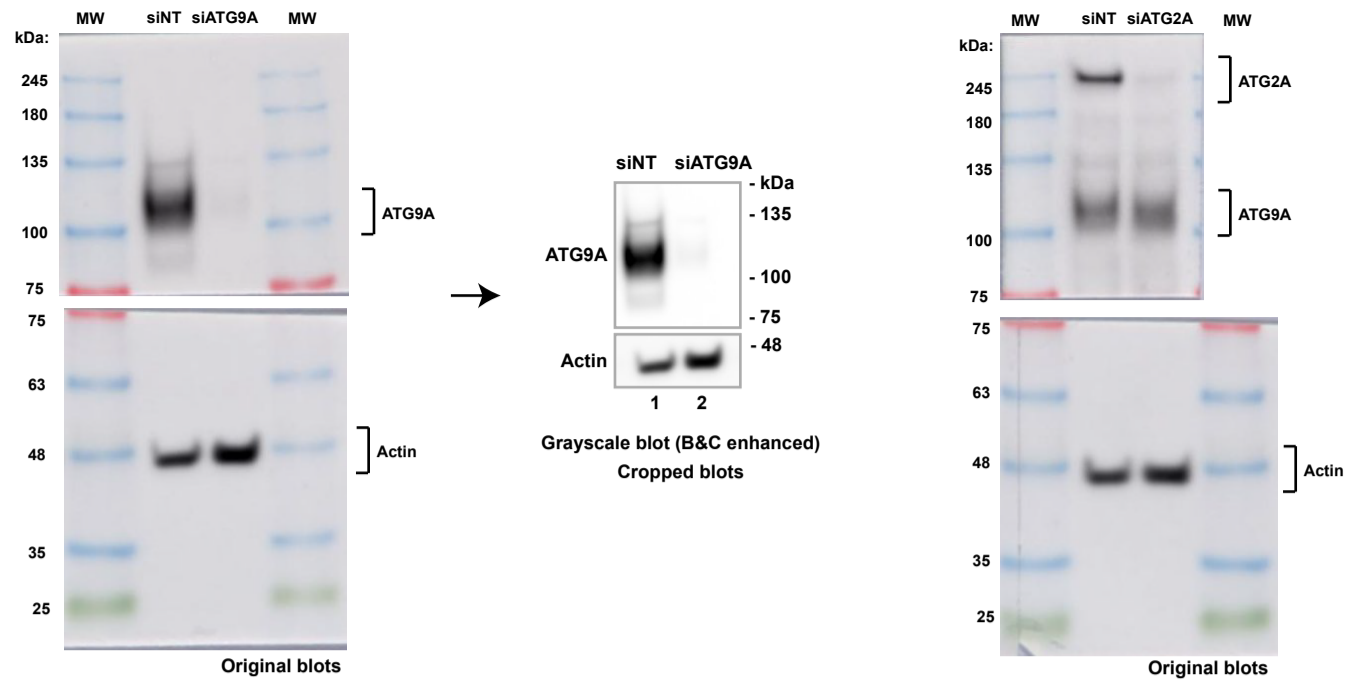

Fig 3G

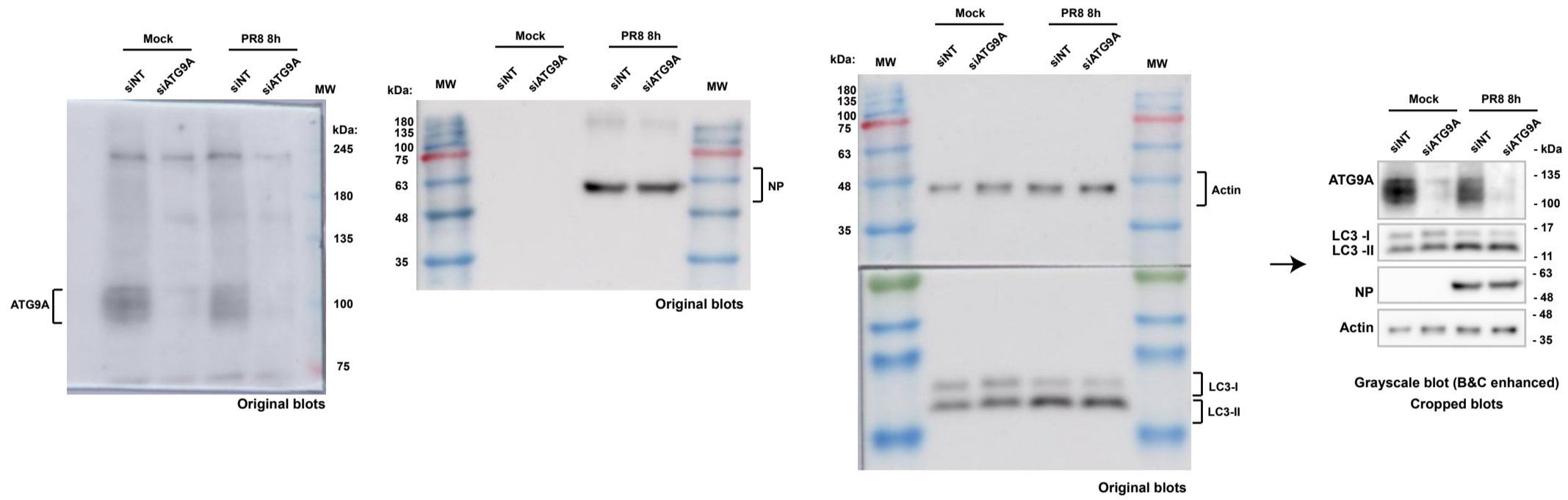

Fig 4C

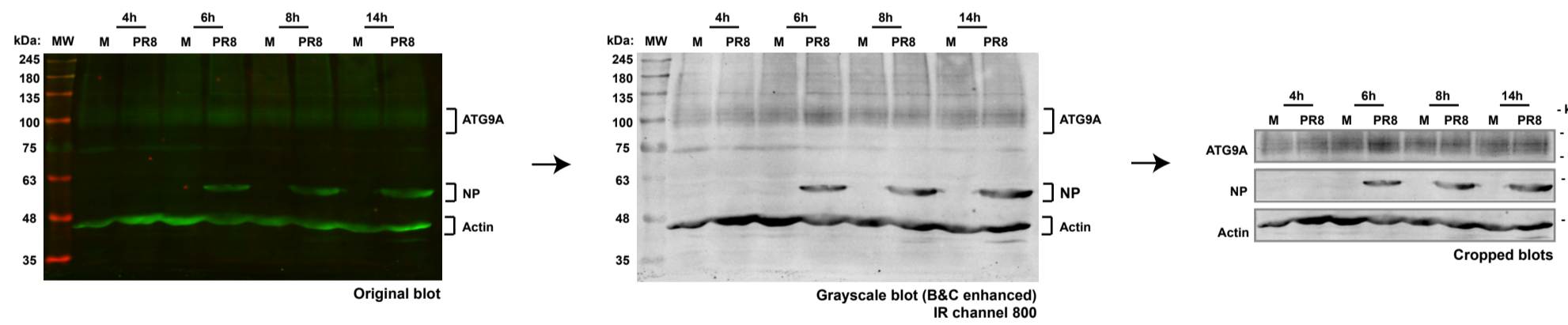

Fig 5B

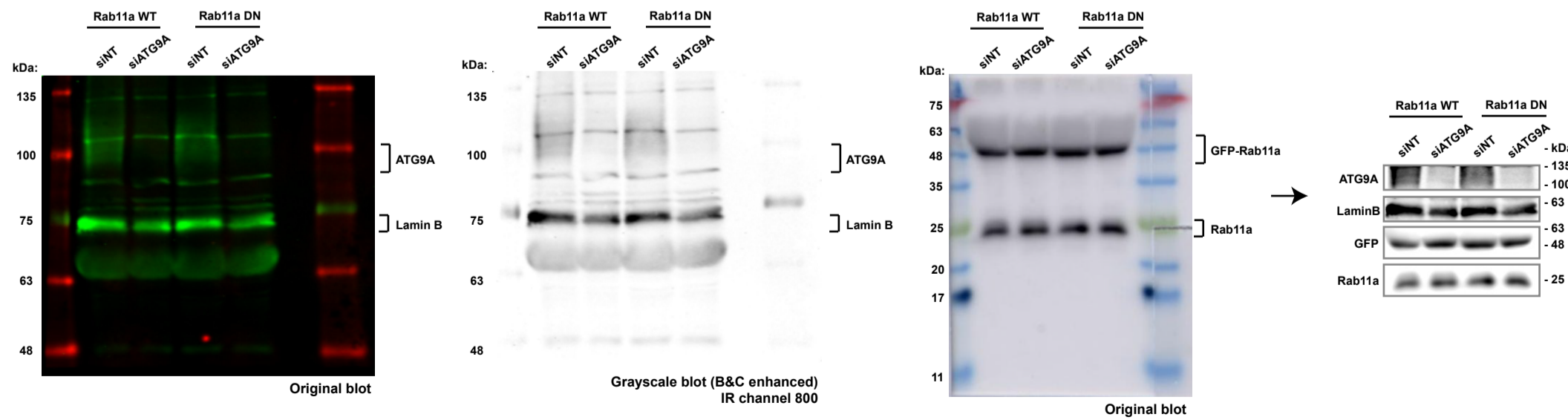

Supplement: S1 Raw Images — Uncropped original blots used in this study are shown. Proteins were detected using the Odyssey infrared (IR) system (green channel, 800 nm; red channel, 780 nm) or chemiluminescence Amersham system. In some cases, original blots were converted to grayscale using ImageJ (FIJI, NIH) and the brightness and contrast (B&C) was adjusted as shown for each case. The final crops shown can be found in the main text in Figs 3C, 3G, 4C and 5B, respectively. The molecular weights of the ladder in kiloDaltons (kDa) are shown for each blot. (PDF) [file pbio.3002290.s008.pdf]
