# Supplementary material for: ATG9A regulates the dissociation of recycling endosomes from microtubules to form liquid influenza A virus inclusions
Source: PLoS Biol. 2023 Nov 20;21(11):e3002290. doi: 10.1371/journal.pbio.3002290 (PMC10695400; doi:10.1371/journal.pbio.3002290)
Supplement: Attachment — Submitted filename: Response to Reviewers.pdf [file pbio.3002290.s028.pdf]

Response to Reviewers: previous interaction [PBIOLOGY-D-22-02738R1]

We would like to express our gratitude to the reviewers and editors for taking the time to evaluate our manuscript "ATG9A regulates dissociation of recycling endosomes from microtubules leading to formation of influenza A virus liquid condensates". Three primary concerns have been raised by the senior editor and the reviewers, concerning:

1. The significance of ATG9A to the formation and function of influenza A virus (IAV) liquid viral inclusions.
2. Whether ATG9A involvement in such a process is related to autophagy.
3. The seemingly contradictory presence of membranes in membraneless viral inclusions, while also defining the nature of Rab11a-endosomes in these condensates.

While we appreciate and agree with the first and second points of the assessment, and have addressed them as proposed below, we believe that the third critique may be due to a lack of familiarity with our system. This indicates that we overlooked important information necessary to address the perceived inconsistencies with previously published work, including our own, and we have included it in the revised version. This last point is so important that we decided to clarify it before explaining our modifications for revisions.

The objective of this study is to enhance our understanding of the development of IAV liquid inclusions that arise during infection via viral modulation of the host recycling endosome. It is worth noting that the formation of numerous liquid condensates (stress granules, Tiger, autophagy, or Sec bodies formation) in the cell is initiated in response to specific stimuli [1,2]. Therefore, our study has broader implications for biological systems by demonstrating the flexibility of cellular machinery to change function and give rise to biomolecular condensates. Here we show that ATG9A is a modulator of liquid-liquid phase separation on or near the ER in mammalian cells, which has also been recently proposed by another group [3].

In fact, IAV inclusions are a liquid condensate formed by the aggregation of Rab11a recycling endosomes [4], similarly to the synaptic vesicle clusters formed in neurons by phase separation for their ready deployment upon synaptic stimuli [5]. Viral inclusions share properties with *bona fide* liquid condensates [6], meaning they are dynamic, react to stimuli and internally rearrange. Moreover, although viral inclusions contain membranes inside, they are not delimited by a membrane like classical membrane-bound organelles (Figure 1 below) [4,7]. Both Rab11a endosomes and vRNPs co-localize in these condensates [4,7]. Our proposed model is that viral progeny RNA segments (vRNPs) bind to Rab11a-GTP on Rab11a recycling endosomes during IAV infection [8,9], and rather than being co-transported to the plasma membrane, they accumulate close to ER exit sites (ERES) [4]. As a consequence, the Rab11a-regulated recycling activity is impaired by IAV infection [10,11].

In our model, the liquid-like character results from a network of weakly interacting vRNPs that bridge multiple cognate vRNP-Rab11a units on flexible membranes [7]. This has important consequences on how the IAV genomic complexes are formed, which are composed of 8 different RNA segments, and constitutes a change in paradigm.

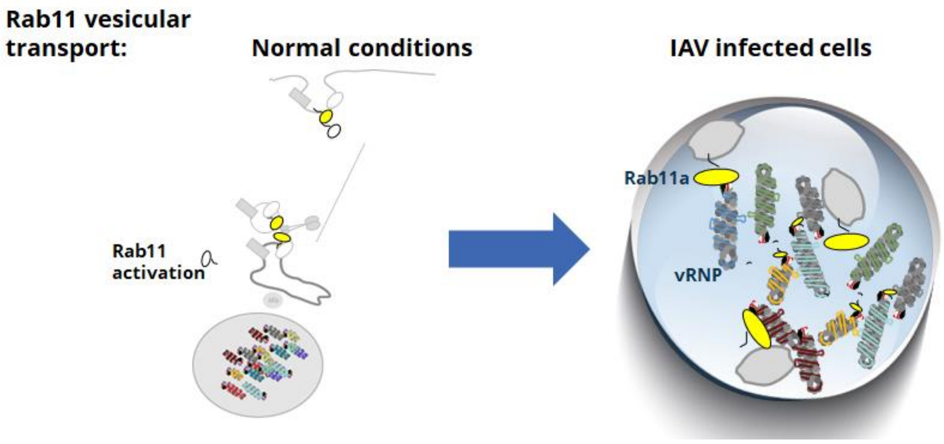

Figure 1. IAV inclusions have liquid properties and are viewed as sites for genome assembly.

In normal conditions, Rab11a endosomes are able to recycle cargo to the plasma membrane using the microtubule network. Upon IAV infection, vRNPs bind Rab11a (via PB2 viral protein) and as the cytoplasmic content of vRNPs increases [8,12–15], vRNPs outcompete Rab11a adaptors/molecular

motors for Rab11a binding [8–10]. As a consequence, recycling is impaired during infection [10] and Rab11a endosomes bound to vRNPs are re-routed to ERES to form viral inclusions that display the properties of liquid condensates [4,7].

Data supporting our proposed model includes:

1. Progeny vRNPs utilize Rab11a endosomes by binding to Rab11a facing the cytosol (not being inside) [8,12–15].
2. Rab11a forms enlarged cytosolic puncta in infected cells, where the different vRNPs concentrate [8,12–15]. This raised the question of whether they contained complete IAV genomic complexes and these structures were named **viral inclusions** [4].
3. However, we found that viral inclusions formed when one vRNP type was expressed in cells [4]. This led to the proposal that these are sites dedicated to the assembly of the IAV genomic complexes, each composed of 8 different vRNPs.
4. Blocking the formation of IAV inclusions, by many distinct processes, reduces viral production [7].
5. In IAV infection, Rab11a positive vesicles accumulate close to ERES [4,10].
6. During IAV infection, Rab11a-regulated endocytic recycling is impaired [10,11].
7. Whilst vRNP binding to Rab11a was proposed in 2012 to play a role in transporting vRNPs to assembly sites at the plasma membrane using microtubules [8,12–15], it was later refined. Many groups, including us, found that the Rab11a endocytic recycling activity was lower during IAV infection on account of reduced microtubule related movement [10,11,16].
8. Although several groups demonstrated that vRNP binding to Rab11a outcompeted that of molecular motors, a recent study challenged this view and suggested that rather than outcompeting, the process would be regulated [16].

9. Both Rab11a and vRNPs drive the formation of viral inclusions [4,17].
10. We showed that viral inclusions have properties of liquids and that hardening them impairs viral fitness, revealing that their material properties are key for infection [4,7].

In this study, we confirmed that Rab11a endosomes carrying vRNPs derail from microtubules and accumulate near the ER to form functional liquid viral inclusions. We show, for the first time, that this process can be reversed by reducing ATG9A levels, meaning that it can be controlled. ATG9A depletion provokes changes in the morphology and behavior of IAV inclusions, and this has a negative impact in IAV viral production. We propose that the amount of complete viral genomes formed inside the cell is reduced, leading to lower levels of virions released. This further strengthens that for optimal function, viral inclusions require a certain shape and cellular localisation. While ATG9A reduction has only a minor impact on viral production (0.6 log), its simultaneous depletion with Rab11a results in a significant inhibition of viral production by 3 logs of magnitude. These findings highlight the independent yet synergic role of both pathways in IAV infection.

For clarity, we divided this proposal to address reviewers' comments in two additional sections, one where experiments to address the three major concerns are explained and another consisting of a point-by-point response to each reviewer.

Proposed experiments to address the reviewers major concerns

We propose to re-submit the manuscript addressing the points that all three reviewers raise and that are listed below.

1) The significance of ATG9A to the formation and function of IAV liquid viral inclusions.

In this manuscript we proposed to address ATG9A significance and changes in liquid condensate shape to IAV production. We show that in the absence of ATG9A there are less virions produced, but they contain similar amounts of complete genomes as the controls. This does not mean that the environment to create complete genomes inside viral inclusions is not compromised when ATG9A is absent and less complete genomes are produced. In the revised version, we quantified the amount of all viral RNA segments (1 to 8) inside the cells by RT-qPCR under the two conditions (siNT vs siATG9A), to explore if more vRNPs remain in the cytosol, which could indicate less viral genomes produced. We observed that vRNA levels for the 8 segments were increased in cells depleted of ATG9A (2-3 fold increase), indicating that there was an accumulation of vRNPs in the cytosol.

2) Whether ATG9A involvement in such a process is related to autophagy.

We will clarify if the role of ATG9A in IAV infection is related to autophagy or not. To address this point, we have performed the following experiments:

1. We performed a small siRNA screening to test if autophagy factors were key for viral production and for viral inclusion development, namely those involved in canonical autophagy initiation (ULK1 and ULK2, Unc-51 like autophagy activating kinase 1/2), in membrane remodeling for autophagosome formation (ATG2A and ATG9A, autophagy related gene 2A and 9A) or in the negative regulation of membrane delivery from Rab11-recycling endosomes to forming autophagosomes (TBC1D14, Tre2/Bub2/Cdc16 1 domain-containing protein 14). We observed that only ATG9A

caused both a drop in viral production and induced tubulation of vRNP-Rab11a endosomes.

2. We detected lipidation of LC3, the hallmark of autophagy, on cells treated with or without siATG9A and infected or mock-infected with IAV. We could not observe any statistically significant differences in the levels of LC3-II in all tested conditions, suggesting that the effect of ATG9A in viral inclusions is unrelated to activation of the full autophagy pathway. Of note, LC3 lipidation occurs during IAV infection, as demonstrated by [18]. However, LC3 binds to a LIR motif on M2 and is relocated to the plasma membrane, rather than concentrating on autophagosomes [18].

3) The seemingly contradictory presence of membranes in membraneless viral inclusions, while also defining the nature of Rab11a-endosomes in these condensates.

We better explained in the Introduction and Discussion sections of the manuscript how we conciliate our previous findings that liquid viral inclusions are not delimited by a membrane like conventional organelles (ER, mitochondria, peroxisomes, etc) [4,6,10,17], and yet contain membranes inside (this was explained above).

Point-by-point response to reviewer remarks (in blue):

When feasible, we provided a point-by-point response to each reviewer.

Reviewer #1: An important step in Influenza A virus (IAV) biogenesis is the coalescing of new viral genomes and viral NP protein in condensates in the cytoplasm. These condensates have previously been shown to form at ER exit sites, move along microtubules, and contain Rab11 - a small GTPase that is normally associated with recycling endosomes. In this paper, Vale-Costa report that the autophagy protein ATG9A plays a role in condensate dynamics. ATG9A is a lipid scramblase that is thought to facilitate the growth of the phagophore membrane in autophagy, in addition to having some seemingly non-autophagic functions. The effect of ATG9A on IAV replication is not dramatic, but the cell biology is still interesting. The core finding in this manuscript, in my opinion, is the altered appearance and dynamics of IAV condensates upon siRNA depletion of ATG9A. The condensates become drawn out, and the authors use beautiful live-cell imaging to show that this is due to increased association with microtubules. Unfortunately, the manuscript lacks in explanations for this observation, and even the justification for studying ATG9A is missing. I can thus not recommend publication unless these major concerns can be addressed. Since they are likely to require a lot of time and have uncertain outcome, I also understand if the editor cannot invite a revision.

Major concern 1:

On line 187-8, the authors write "From an siRNA screening of host factors impacting IAV infection, ATG9A stood out as a putative candidate to explain viral inclusion formation". However, they neither show any screening data themselves, nor do the references at the end of that sentence (33-35) contain any siRNA screens for IAV replication. I am aware of one paper that did screen autophagy proteins for roles in virus replication, including IAV (PMID: 27573464). However, that paper identified several ATG proteins as being similarly important to ATG9. The authors must properly explain their reasoning for studying ATG9. If they are

leaning on the paper I mentioned, I would strongly suggest to repeat some of the key experiments (microtubule association of condensates) with some of the other top ATG hits from the screen. At a minimum, I would suggest to look at ULK1/2 (the initiators of canonical autophagy, for which there are small molecule inhibitors), and ATG2, which works in a pair with ATG9 in autophagy.

We have performed a small siRNA screening (Fig 3A-F of new manuscript version) to test the effect of depleting ULK1/2, TBC1D14 and ATG2A in infected or mock-infected cells to determine viral production (by plaque assay) and liquid viral inclusion formation (immunofluorescence). As no differences in both the previous parameters were identified upon depletion of these autophagy factors (and no tubulation phenotype observed in viral inclusions), we did not test further the association of viral inclusions to microtubules in these cases.

Major concern 2:

The authors should do some standard autophagy assay (I would suggest LC3 lipidation) on their cells +/- infection and upon ATG9 KD. The discussion of "non-canonical" function of ATG9 cannot be properly held without seeing if autophagy is induced.

As suggested, we detected lipidation of LC3, the hallmark of autophagy, on cells treated with siNT or siATG9A and infected or mock-infected with IAV (by western blotting, Fig 3G-I of new manuscript version). No differences were observed in LC3 lipidation levels upon ATG9A depletion. Of note, LC3 lipidation occurs during IAV infection, as demonstrated by [18]. However, LC3 binds to a LIR motif on M2 and is relocated to the plasma membrane, rather than concentrating on autophagosomes [18]. For these reasons, we have concluded that the ATG9A role in our system is unrelated to the autophagy pathway.

Major concern 3:

The paper lacks mechanistic detail as to how ATG9 might have its effect on IAV condensates. The fluorescence microscopy is largely well made, but the kind of proximity of fluorescence signals shown in Fig. 3F is not a proof of anything. The authors should provide more insights into how ATG9 is involved. One example of such insights would be convincing proof of interaction between ATG9 and some viral proteins.

Given that viral inclusions are formed via phase separation, physical interactions between proteins cannot be detected, hence why we decided to observe interactions by immunofluorescence. However, the type of colocalization we see between ATG9A and viral inclusions is modest and in our model ATG9A may respond to a cellular signal, leading to reorganization of Rab11a endocytic recycling. This does not necessarily mean that ATG9A and Rab11a must colocalize.

Major concern 4:

I cannot wrap my head around how ATG9 depletion leads to less virus release when MORE viral proteins are found at the plasma membrane (Fig. 6A) and trafficking of inclusions in the cytoplasm is FASTER (Fig. 5H). I would have expected it to lead to more virus release. At a minimum, this apparent conundrum must be discussed thoroughly.

This is exactly the point of our paper, and thank you for asking for the clarification. We aim to demonstrate that having tubular viral inclusions that move on microtubules rather than having rounded viral inclusions lodged close to ERES, reduces the formation of complete genomes inside the cell. Detaching from microtubules to accumulate near the ERES produces optimal viral inclusions that are able to assemble complete 8-vRNP genomes, unlike the tubular inclusions that are attached to microtubules. We have removed the phrasing “FASTER” from the manuscript as that did not accurately describe the effect observed. The tubular inclusions are able to displace more because they are continuously moving in microtubules, not necessarily that they move faster. In addition, we aim to show that no other step in the viral

replication cycle is involved. Supporting data include the demonstration that (Fig 7A-F of new manuscript version):

1. the number of released virions is lower when ATG9A is absent and viral inclusions are tubular;
2. the progeny virions released from ATG9A depleted cells still contain complete genomes;
3. the amount of viral transmembrane proteins at the plasma membrane increases.

This is consistent with a blockade in viral assembly distinct from viral transmembrane proteins production and transport, and points towards a defect in the efficiency of complete genomes being formed inside the cell. The accumulation of transmembrane proteins at the plasma membrane results from reduced budding and release of viruses.

Major concern 5:

Quantifications of data (microscopy images, blots etc) have not been done consistently. A key example is the missing analysis of correlation between microtubule and Rab11 staining in Fig. 5. This should also be quantitated with NP. A quantitation of the amount of remaining ATG9 protein after siRNA KD should also be performed, e.g. on western blots, to complement the mRNA measurement. Also, there are several cases of mean and std calculated from two datapoints (e.g. Fig. 6A). Three should be the minimum.

We have performed consistent quantifications of data as suggested.

Some other points:

1. The serial tomograms are impressive but only one each of them is shown, with no quantifications of features. If the legend is correct in saying that >10 cells were imaged, some quantifications of the tomograms should be possible, or at a minimum snapshots of other

tomograms could be shown in a supplementary figure. In either case, the small number of tomograms means that discussions based on them should be made more cautiously. As a side, in figure 1A, a height in nanometres would be more informative than section number.

We have performed quantifications of the number of single and double membrane vesicles throughout infection (Fig 2E-G of new manuscript version). We included this in the manuscript along with the serial tomograms. We have also included the section height in Fig 2C, 2D and S2A Fig (of the new manuscript version), as suggested. Figure legend now reads: “Representative cells are shown with 3 individual sections **(including section height in nm)** and the 3D cumulative model.”

2. "vacuole" in Fig 1B should not be named such. That is a plant and yeast term. This might be an endosome or even an invagination of the PM. If you want to suggest it is a dilation of the ER, call it "ER dilation", but it should be clear that that is just one possible identity.

We have changed “vacuole” to “ER dilation” in the main text, in Fig2 and S2 Fig (new manuscript version).

3. Fig 1D: some images seem swapped. E.g. the 0208 and 0252 Rab11a panels don't match the overlay.

Thank you for pointing that out. The order of the swapped crops has been corrected.

4. Line 59: "containing no limiting membrane" is not a "liquid property".

We corrected this.

5. Line 88: The statement "Whether the ER plays a role in the biogenesis and dynamics of IAV inclusions and which cellular factors regulate their phase separation at the ER is unknown" seems to broad to me given the already published work by the authors and others.

We introduced substantial modifications to the introduction as it is as clear to us that it really did not convey the necessary background data.

6. Line 121 etc: The standard abbreviation of electron tomography is ET. Please avoid ET-TEM.

ET-TEM was used to abbreviate electron tomography transmission electron microscopy. To avoid confusion, we have kept the "ET" abbreviation for electron tomography and "TEM" for electron transmission microscopy. We have changed this as follows: " (...) we resolved the 3-dimensional (3D) organization of viral inclusions by high-pressure freezing / freeze substitution and electron tomography (ET) transmission electron microscopy (TEM)".

7. Line 177: should it not be Fig. 1D?

We corrected this.

8. Line 200 and Fig. 2B: the only way all measurements of siNT could be exactly 1.0000 is if they were normalised to that. In that case, showing this bar is pointless, just show the siATG9A and say that it was normalised to siNT.

We understand the reviewer point of view, but it is not considered incorrect to plot the results in this way and we feel that it is more obvious for the reader to read the data like that, as we have done in past publications. If reviewer 1 is very against it, we will change it.

9. Line 203-4: I find the statement "we could not detect ATG9A subcellular localization in infected cells, which could be due to protein relocalization to unknown sites" odd. I suggest the authors instead say what they did see: a cytoplasmic (possibly perinuclear) staining which no longer colocalises with the Golgi.

We have changed our statement as suggested: “We observed that ATG9A presented a cytoplasmic (possibly perinuclear) staining in infected cells which no longer colocalised with the Golgi”.

10. Fig. 2D does not show good examples of "roundness vs circularity".

To better illustrate the changes in circularity and roundness of viral inclusions, we have also included the following scheme in Fig 3E (new manuscript version):

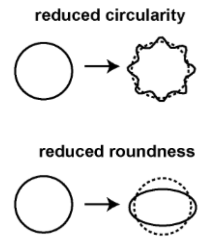

11. Fig. 3C would benefit from showing a slightly bigger crop and also from a quantitation of triplicates.

We have already done this modification and quantifications, which were included in updated Fig 4C (Fig 3C in previous version).

12. Line 312-3: The expression "significantly decreases" requires a quantification.

We have changed the expression “significantly decreases” to “is lost with”, as follows: “We first confirmed that GFP-ATG9A has a strong localization at the Golgi in mock-infected cells, which is lost with infection (Fig 3D).”.

13. Line 314: I would be not use the word "interaction" since that may be understood as a direct protein-protein or protein-RNA interaction. The fluorescence microscopy used cannot establish that.

We have removed the word “interaction” and kept “multiple contacts”, as follows: “We also observed that GFP-ATG9A could establish multiple contacts with viral inclusions (...)”. phase separation transient interactions difficult to observe in pull down assays

14. Line 316: The description "viral inclusions seemed to be associated with the ER through ATG9A puncta" is too speculative. It could be replaced by something along the lines of "found in the vicinity of".

We have corrected this as suggested. It now reads: “Moreover, ATG9A puncta was found in the vicinity of viral inclusions and the ER (inset in Fig 4F).”

15. Fig. 5: The title should be "on" microtubules. Otherwise, the reader may think it is "in" the lumen of the microtubules.

We have replaced “in” for “on” as suggested.

16. For Fig. 5I, can the motility be quantified as done in 5H?

We have performed quantification of the mean squared displacement (MSD) for cells depleted of ATG9A treated with or without nocodazole, which is now shown in Fig 6 of new manuscript version.

17. Figure 6A doesn't show a "gating strategy" as claimed on line 474. Also, it is not clear which protein is measured in 6A.

The expression “gating strategy” was removed. The first plot was a representative schematics of how the data was acquired by flow cytometry. To avoid confusion, we have removed this plot and kept only the Mean Fluorescence Quantification bar charts. We have indicated clearly in the figure legend and at the top of each MFI plot which protein is being measured.

“The levels of the three viral surface proteins at 8h p.i. (HA, hemagglutinin; NA, neuraminidase; M2, Matrix protein 2) were determined by flow cytometry using monoclonal antibodies against each viral protein and analyzed as shown.”

18. Fig. 6B: the quantifications should be done on triplicates.

We have repeated the experiment 2 more times to increase the number of experimental replicates, so now the data corresponds to a pool of 4 independent experiments (Fig 7C of new manuscript version).

19. Line 554: The authors cannot claim that the function is "beyond its well-known involvement in autophagy" unless they have done autophagy assays etc. See major 2.

To address this point, as proposed by reviewers #1 and #3 (results in new Fig 3A-F):

1) We have performed a small siRNA screening to test the effect of depleting ULK1/2, TBC1D14 and ATG2A in infected or mock-infected cells to determine viral production (by plaque assay) and liquid viral inclusion formation (immunofluorescence). As no differences in both the previous parameters were identified (and no tubulation phenotype observed in viral inclusions), we did not test further the association of viral inclusions to microtubules.

2) As suggested, we detected lipidation of LC3, the hallmark of autophagy, on cells treated with siNT or siATG9A and infected or mock-infected with IAV (by western blotting). No differences were observed in LC3 lipidation levels upon ATG9A depletion. Of note, LC3 lipidation occurs during IAV infection, as demonstrated by [18]. However, LC3 binds to a LIR motif on M2 and is relocated to the plasma membrane, rather than concentrating on autophagosomes [18].

20. Line 571-2: I don't see how the competition can be ruled out as being irrelevant.

We will rephrase this part. Many groups demonstrated that vRNP binding to Rab11a outcompeted that of molecular motors [8–10] although a recent study challenged this view and suggested that rather than outcompeting, the process would be regulated [16]. In the discussion of the new manuscript version, it now reads:

“Still in both cases, this work shows that in the context of infection, the dissociation of viral inclusions from microtubules is a regulated process, as vRNPs bound to Rab11A can be placed in microtubules when ATG9A is absent. This strongly suggests that the competition between vRNPs and molecular motors for binding to Rab11a, as initially proposed by us and others may be a part of the process but is unable to explain the unbinding of Rab11a vesicles from microtubules fully.”

21. Line 625: MDCK are not human cells.

We thank the reviewer for spotting this mistake. The term human was incorrect and was removed from the sentence.

Reviewer #2: This is a follow up to previous work from this laboratory demonstrating assembly of IAV vRNPs in viral inclusions made from liquid organelles (LO) that form close to the ER at ER exit sites. These liquid organelles recruit Rab 11 which is important for recycling proteins from endosomes to the plasma membrane. Liquid organelles also exchange material through fusion and fission events that require microtubules. Previous correlative light EM suggested that the liquid organelles formed during IAV infection lack a delimiting membrane, but the LOs do require ER-Golgi trafficking because they are lost when cells are incubated with brefeldin-A or following disruption of ER exit sites by inhibiting the SAR1 GTPase. The current study uses high quality microscopy to look at interactions with the ER more closely and provides evidence that ATG9A, a protein required for autophagy, may modulate tripart interactions between viral inclusions/LOs and the ER and microtubules. ATG9A is a scramblase that flips phospholipids between membrane leaflets during expansion of autophagosomes. Interestingly, ATG9A can also mobilise transfer of lipids to lipid droplets, which share properties with liquid organelles.

The first experiments describe the use of high pressure freezing and EM tomography to provide 3D reconstructions of ER:LO interface. New double membraned vesicles, single membrane vesicles and enlarged ER lumen are seen close to the ER. It is not clear how this relates to LOs that do not have a limiting membrane.

Liquid viral inclusions do not have a delimiting membrane, although they do contain membranes on the inside, either single or double membrane vesicles. These aggregates develop close to the ER. What we propose is that the ER may support the development of liquid viral inclusions. In fact, membrane surfaces are emerging as key sites for the regulated assembly of biomolecular condensates including Tiger and Whi3 RNPs [19]. Membrane surfaces limit the diffusion of proteins/RNA to two dimensions, reducing the minimum concentration of material required for phase separation to take place (reviewed in [19]). We

have included this information in the manuscript (introduction and discussion sections) to help understand our proposed model for viral inclusion formation at the ER.

Similarly, immunogold labelling is used to study co-localisation of Rab11 and NP double labelling is seen on single membrane vesicles (green), double membrane vesicles (yellow) and ER (blue). The text ln163 says 'It is noteworthy that numerous vRNPs detected inside viral inclusions were not attached to any membrane (Fig 1C). These are not indicated in the figure. At this point the data suggest that Rab11 is a marker for membrane compartments, rather than LO.

IAV liquid inclusions are an LO formed by aggregation of vesicles, similar to what happens in synaptic vesicles in neurons ready for deployment upon stimuli [5]. The accumulated vesicles are brought together by a mechanism similar to proposed for other LOs, and are not delimited by a membrane as classical membrane organelles [1,6]. As both Rab11a vesicles and vRNPs co-localize in these condensates, they can be interchangeably used as LO/viral inclusions markers [4,8,10,17]. Here, we used the immunogold labeling to demonstrate that IAV LOs/viral inclusions are in close association with the ER, contain vRNPs bound to Rab11a on membranes, and vRNPs that are bridging these structures (S2B Fig in the new manuscript version). We have better explained this in the introduction of our manuscript. Our model is shown in Figure 1 below. We have removed the sentence about vRNPs unbound to membranes in the text as it may cause confusion.

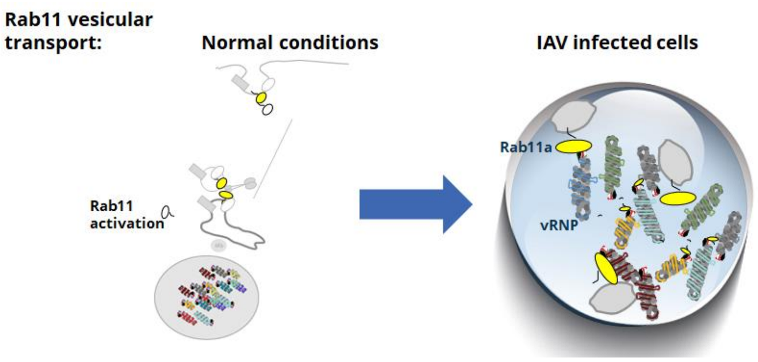

Figure 1. IAV inclusions have the properties of liquids.

In normal conditions, Rab11a vesicles are able to recycle cargo to the plasma membrane using the microtubule network. Upon IAV infection, vRNPs bind Rab11a (via PB2 viral protein) and as the cytoplasmic content of vRNPs increases [8,12–15], vRNPs outcompete Rab11a adaptors/molecular motors for Rab11a binding [8–10]. As a consequence, recycling is impaired during infection [10] and Rab11a vesicles bound to vRNPs are re-routed to ER exit sites to form viral inclusions that display the properties of liquid condensates [4,7].

Live cell imaging of Rab11a (as a marker for LO or recycling endosomes?) and fluorescent ER marker looks at interactions between Rab11 and the ER. In mock infected cells Rab11 is located to tubules while Rab11 is seen in rounded structures after infection with IAV. A line scan follows fusion of LO with the ER in infected cells but does not follow interaction of Rab11 with ER in uninfected cells. The videos are difficult to interpret. A line scan for interaction in mock infected cells should be provided to support the main conclusions that ER contacts are increased for Rab11 after infection.

We have performed the line scan quantification for mock infected cells and included it in the manuscript along with similar data for infected cells (Fig 1E in the new manuscript version). Please consider that Rab11a in mocks is a small structure in the cell whilst Rab11a in infected cells results from accumulated vesicles and hence is a large structure.

Silencing of ATG9A produces a small drop in viral titre and converts LO into tubular networks and appears to reduce association of LO with ER. Here the virus is tracked with antibodies to NP. Some explanation for why the experiments track NP, rather than Rab11, is needed.

We have tracked LOs/viral inclusions with both NP and Rab11a whenever possible and for simplicity the results may have been shown in distinct figures. Nevertheless, for circularity and roundness quantifications, we prefer segmenting viral inclusions using NP antibody staining as it produces higher signal-to-noise ratio when compared to Rab11a antibody staining (Fig 3F and S3B Fig in the updated manuscript version).

LO contacts are reduced following silencing of ATG9A. The quantification is difficult to follow because arrows in figure 2E and 2F indicate different events making comparison difficult. Is the conclusion based on a visual interpretation where rounded LO are easier to image than tubular LO, or it is easier to surround a round LO rather than a tube, without making contacts?

In the case of cells depleted of ATG9A, rounded LOs are not formed. Rather, tubular shaped structures are formed that contain Rab11a vesicles carrying vRNPs being transported by microtubules. We have quantified the shape of the structures obtained in Fig 3 (Fig 2 in previous version). In Fig 2E of the previous version, we aimed to show that these differences in the shape of LOs give rise to different numbers of fusion and fission events. However, we realized that in the current Fig 6, with the live cell imaging and calculations of mean squared displacement, we can assess the dynamics of viral inclusions and we have removed former Fig 2E from the manuscript.

Later in the paper the effects of DN Rab11 are explored (Figure 4). The DN Rab11 produced a large drop in virus titre which was further reduced by silencing ATG9A. The DN Rab11 is not studied further so the role of Rab11 remains unclear. The distribution of vRNPs and Rab11 positive recycling vesicles is described, (4D) but if these vesicles (recycling endosomes?) have membranes they cannot be the liquid organelles that are the focus of the study.

The Rab11a DN was not further explored as we concluded from those experiments that the effect of ATG9A on infection was not related to ATG9A direct modification of the Rab11a recycling endosomes (Fig 5 in the new version). As the same drop in viral production is obtained upon ATG9A depletion in both Rab11a WT and DN cells, ATG9A effect must be independent of Rab11a-regulated recycling role and that is what we aimed to demonstrate with this experiment.

Recent evidence demonstrates that phase separated condensates can contain vesicles in their constitution as mentioned in the second comment by this reviewer. We hope that now this matter has been clarified.

The paper concludes " that ATG9A is critical for proper establishment of IAV inclusions at the ER but is unlikely to be mobilized from the recycling endosome nor does it influence the association of vRNPs to Rab11a vesicles.'" This is difficult to follow because the images do not look at the ER or the effect of loss of recycling endosomes following expression of DN Rab11.

Our data indicates that the role of ATG9A and of Rab11a are independent. This is supported by data on titres in Rab11a DN cell lines that produce a viral drop that is 0.5 log when ATG9A is depleted, which is the same value obtained for Rab11 WT ATG9A depleted cells. Our model is that ATG9A may respond to a cellular signal, leading to reorganization of the Rab11a endocytic recycling to ER exit sites. This does not necessarily mean that ATG9A and Rab11a must colocalize.

Of note, in cells Rab11a DN the progeny vRNPs exported from the nucleus do not bind Rab11a DN and are not able to aggregate into LOs/viral inclusions, thus becoming scattered in the cytoplasm [4,8,10,17]. This is why we cannot follow Rab11a-vRNP association in Rab11 DN cells. However, show in the current manuscript how the Rab11a-regulated recycling proceeds in Rab11a WT and DN in mock and infected cells (Fig 1B-C) and we have also included immunofluorescence images of how vRNP distribution looks like in Rab11 DN cells (S1 Fig and Fig 5C of updated manuscript version).

The last part provides convincing evidence that the shape of Rab11 positive structures is determined by association with microtubules. Again the text refers to Rab11 vesicles which

most cell biologists would think are recycling endosomes rather than liquid organelles. At other times they are called viral inclusions, but are these liquid organelles?

We know that during IAV infection, endocytic recycling is impaired [10], and that Rab11a positive vesicles accumulate close to ER exit sites when Rab11a binds vRNPs. We also demonstrated that these accumulated vesicles exhibit features of liquid condensates - fuse and divide, dissolve with hypotonic shock and with hexanediol, undergo internal rearrangements and are highly dynamic [4,17]. We named these structures ***liquid viral inclusions,*** and so we interchangeably use the terms liquid viral inclusions / liquid organelles / liquid condensates. Hence, we proposed that the binding of vRNPs to Rab11a on recycling endosomes alters endocytic recycling function and behavior by a mechanism that is not well understood. We and others have shown *in vitro* that binding of Rab11a to vRNPs competes with the binding of Rab11a-Family Interacting Proteins (FIPs), which is necessary for Rab11a to attract molecular motors and move to the plasma membrane [9–11]. Another study, however, showed that IAV infection impairs the recycling endosome specifically by decreasing dynein recruitment and not the FIPs [16]. This manuscript shows that the model of out-competition is likely to be wrong, as in conditions where Rab11a is bound to vRNPs as in the case of ATG9A depleted cells, Rab11a can still be transported using microtubules.

Finally, it is shown that ATG9A affects the shape of the Rab11 vesicle/viral inclusions but does not affect genome packaging or virus assembly. This provides further confusion because it is at odds with the concluding sentence.

''Taken together, these data suggest that ATG9A is likely involved in the regulation of viral 516 inclusion distribution, facilitating circulation between microtubules and the ER. By interfering with viral inclusion trafficking, viral genome assembly and delivery to budding sites at the plasma membrane may be delayed or decreased, thus causing accumulation of HA, NA and M2 at the surface. ATG9A may thus be a host catalyst that facilitates viral genome assembly''.

We aim to demonstrate that having tubular viral inclusions that move on microtubules rather than having rounded viral inclusions lodged close to ERES, reduces the formation of complete genomes inside the cell. Detaching from microtubules to accumulate near the ERES produces optimal viral inclusions that are able to assemble complete 8-vRNP genomes, unlike the tubular inclusions that are attached to microtubules. We have removed the term “faster” from the manuscript as that did not accurately describe the effect observed. The tubular inclusions are able to displace more because they are continuously moving in microtubules, not necessarily that they move faster. In addition, we aim to show that no other step in the viral replication cycle is involved. Supporting data include the demonstration that (Fig 7A-F of updated manuscript version):

4. the number of released virions is lower when ATG9A is absent and viral inclusions are tubular;
5. the progeny virions released from ATG9A depleted cells still contain complete genomes;
6. the amount of viral transmembrane proteins at the plasma membrane increases.

This is consistent with a blockade in viral assembly distinct from viral transmembrane proteins production and transport, and points towards a defect in the efficiency of complete genomes being formed inside the cell. The accumulation of transmembrane proteins at the plasma membrane results from reduced budding and release of viruses.

Reviewer #3: The manuscript by Vale-Costa et al. presents a new insight into the role of autophagy protein ATG9A in mediating the exchange of influenza A (IAV) viral inclusions between the endoplasmic reticulum (ER) and microtubules. The authors suggest that the absence of ATG9A influences the formation of viral genome complexes, thus limiting the production of infectious virions by arresting viral inclusions at microtubules and preventing their accumulation at the ER, one of the main sites of liquid condensates RNA genome assembly. This work builds upon the authors' recent publication proposing a model of the liquid-liquid phase separation of the viral ribonucleoprotein (vRNPs) complexes as a favorable environment for IAV genome assembly. Overall, the experiments described in the paper are well-designed and executed, and the topic of IAV genome assembly and intracellular traffic is both relevant and novel. However, I found the paper a bit difficult to follow at times and some of the experiments and interpretations could benefit from additional clarification. Here are some major and main points to consider:

1. To further strengthen the conclusions, it would be helpful if the authors could provide evidence that the phenotypes observed are distinct from ATG9A's "canonical" function. In other words, there's no evidence or references from other groups to a role of autophagy in IAV viral assembly or vRNPs intracellular organization discussed in this manuscript. After convincingly showing that the autophagy-related functions of ATG9A have nothing to do with the phenotypes observed in this study, then one can start to judge and contemplate novel functions of that protein.

We have performed a small siRNA screening (Fig 3 of new manuscript version) to test the effect of depleting ULK1/2, TBC1D14 and ATG2A in infected or mock-infected cells to determine viral production (by plaque assay) and liquid viral inclusion formation (immunofluorescence). As no differences in both the previous parameters were identified upon depletion of these autophagy factors (and no tubulation phenotype observed in viral

inclusions), we did not test further the association of viral inclusions to microtubules in these cases.

As suggested by reviewer #1, we detected lipidation of LC3 (new Fig 3G-I), the hallmark of autophagy, on cells treated with siNT or siATG9A and infected or mock-infected with IAV (by western blotting). No differences were observed in LC3 lipidation levels upon ATG9A depletion. Of note, LC3 lipidation occurs during IAV infection, as demonstrated by [18]. However, LC3 binds to a LIR motif on M2 and is relocated to the plasma membrane, rather than concentrating on autophagosomes [18]. For these reasons, we have concluded that the ATG9A role in our system is unrelated to the autophagy pathway.

2. The introduction mentions several players with unknown or non-canonical functions, making it difficult to understand the objective of the manuscript. For example, the role of Rab11A in recycling during infection is not clear, given that the authors suggest a strong interplay between recycling endosomes and the ER in IAV genome assembly but in lines 72-78 is mentioned that there is a change in Rab11A function upon infection that has been "refined", suggesting no role in recycling.

We have formally demonstrated in this manuscript, in Fig 1B-C, that the Rab11a-regulated recycling function is impaired by IAV infection.

3. The concept of a "modified" ER (L 75, 166-167, 463) is unclear in molecular terms and clarification would be appreciated. Similarly, the use of "remodeled ER" in lines 180-181 could be further explained. Is "remodeled ER" the dmv? Or is the "remodeled ER" supporting the liquid viral inclusion which is the actual dmv?

The expression “remodeled ER” comes from a paper published in 2017 by [20]. We now have inspected the ER and found that the double membrane vesicles (dmv) we published before

seem to originate from the ER. We agree that this point is outside the scope of this paper and we will remove mention to ER remodeling during IAV infection.

4. The significance of ATG9A as a hit from "an siRNA screen of host factors acting at the ER that impacted IAV infection" (L 187-188) is not fully explained. If the authors do not want to share the siRNA screen, more details on the screening strategy would be helpful: it would be useful to start considering alternative explanations for the role of ATG9A in IAV infectivity. Furthermore, the conclusion that ATG9A "remodels the ER" based on references [33-35] (L 189-190) needs to be tested and verified; those papers show an effect in recipient organelles or donor organelles different from the ER in ATG9A deficient cells. This is an overreaching conclusion from those papers.

As mentioned above, we showed an siRNA screening of key autophagy proteins that impact viral production and liquid viral condensate formation, as is the case of UKL1/2, TBCD14 and ATG2. We found that depletion of UKL1/2, TBC1D14 and ATG2A does not affect both IAV production or liquid viral inclusion development.

We mentioned that ATG9A "remodels the ER" based on references [33-35]. By that we meant that ATG9A was involved in the formation of autophagosomes from a source such as the ERES. We have better explained this upon performing experiments to show that ATG9A has a role in viral infection unrelated to full autophagy activation (Fig 3 of new manuscript version).

5. The hypothesis to explain the involvement of Rab11A and vRNPs in the proximity of ER exit sites (ERES) (L 104-106) could be more specific and clearer, including an explicit role for Rab11A or ATG9A. With that premise and context, it was quite hard to understand the results of the whole section around Fig 1A-C.

We have better explained the involvement of Rab11a and vRNPs in the proximity of ER exit sites (ERES) in the Introduction section. Experimentally we demonstrated 1) how IAV infection

impacts recycling function and rerouting of Rab11a vesicles to ERES to assemble viral inclusions (Fig 1 of updated version) and 2) made a clearer link to how ATG9A might facilitate this process.

In this study, we confirm that Rab11a vesicles derail from microtubules in the presence of vRNPs during IAV infection [8] to form functional liquid viral inclusions that are condensates of accumulated vesicles [4]. We show, for the first time, that this process can be reversed by reducing ATG9A levels, meaning that it can be controlled. ATG9A depletion provokes changes in the morphology and behavior of IAV inclusions, and this has a negative impact in IAV viral production. As such, we propose that the amount of complete genomes formed inside the cell is reduced, leading to lower levels of virions being produced. This further strengthens that for optimal function, viral inclusions require a certain shape and cellular localisation. While ATG9A reduction has only a minor impact on viral production, its simultaneous depletion with Rab11a results in a significant inhibition of viral production by 3 logs of magnitude (Fig 5A of updated version). These findings highlight the independent yet synergic role of both pathways in IAV infection.

The observation that the dmv structure arises from the ER (L 125) is not well-supported, and the argument for the lack of double membrane vesicles in mock-infected cells (L 127-130) could be strengthened. How many cells and sections did they analyze? Are these autophagosomes (also double membrane organelles)? If yes, then cells should have autophagosomes, in close apposition to the ER and mitochondria like many researchers have previously shown. If not, can you characterize these dmv better to make a connection with viral presence in the host? It would also be helpful to better characterize the dmv in relation to viral presence in the host.

We have removed any argument that DMVs emerge from the ER as we agree with the reviewer that this point is confusing and would deviate the story from ATG9A role in infection.

We have nevertheless included the quantification of the number of SMVs and DMVs in mock-infected and infected cells (scored from 10 distinct cells).

The existence of DMV is what prompted this study, because DMV have similarities with autophagosome formation. Another note is that IAV induces autophagy, but at the same time recruits LC3-II to the plasma membrane, by binding to the viral protein M2 that has a LIR [18]. Hence, having DMV does not necessarily mean that the cells will contain many autophagosomes.

We do know that liquid viral inclusions recruit mitochondria (unpublished), as can be seen in the figures 2 and 3 below. However, we think that including this figure would be outside the scope of this manuscrit.

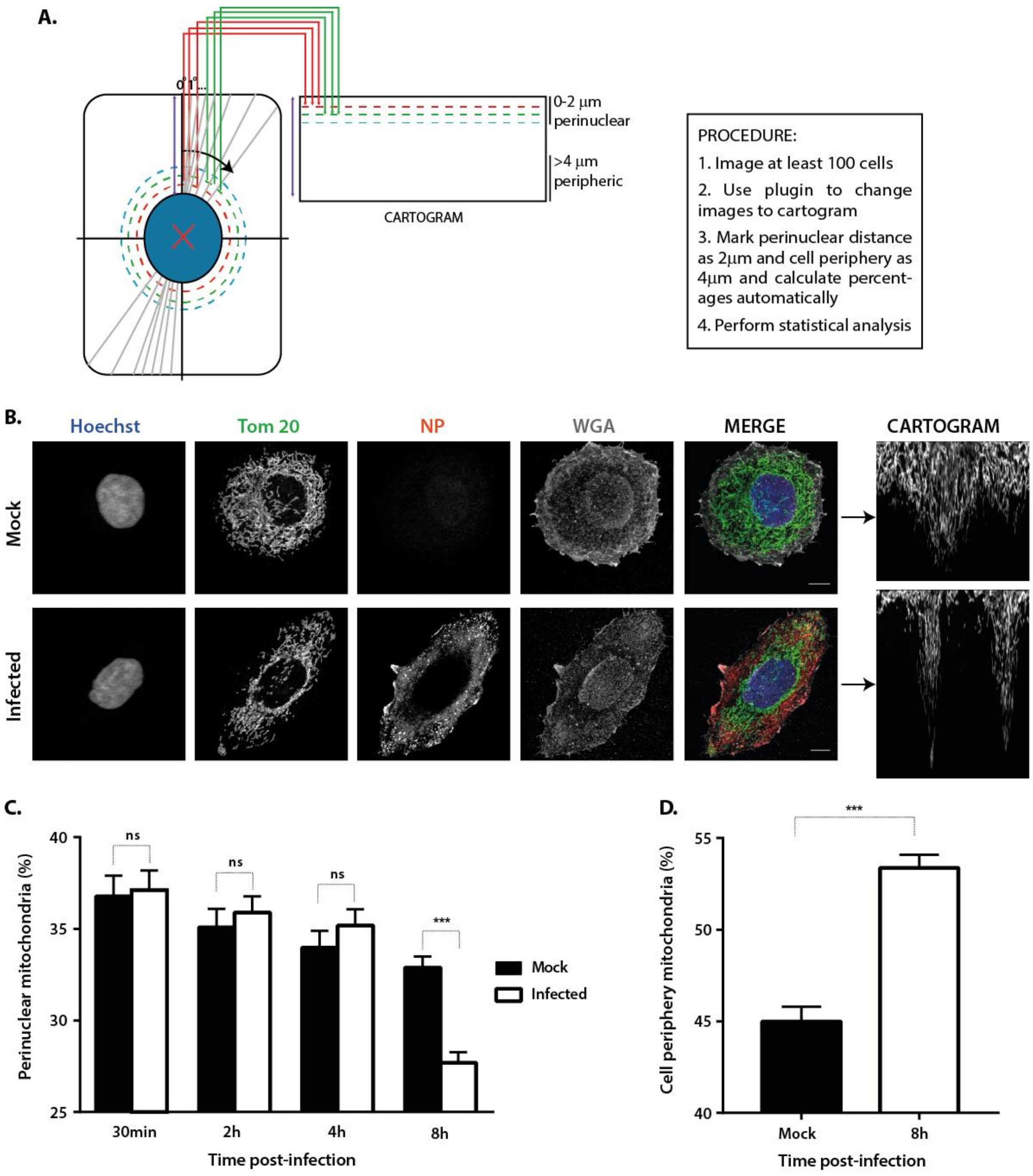

Figure 2 - Systematic single cell analysis of mitochondria distribution during IAV infection.

(A) To quantify the intracellular distribution and concentration of organelles, we have created an automated tool, which consists of the conversion of a confocal image from polar to Cartesian system. Upon establishing the center of the polar coordinate as the periphery of the cell nucleus, and circulating clockwise, we display the signal of each channel one degree apart in a XY graph. The analysis continues repeating the process upon increments of a fixed width, towards the cell surface. The organelle distribution map in this format provides means to accurately quantify polarization, redistribution towards the perinuclear region or expansion to the cell periphery, thus allowing a

systematic analysis. The automated system is powerful because it allows to image and process a statistically significant number of cells using the indicated procedure.

(B), (C) and (D) HeLa cells were infected or mock-infected with PR8, at a MOI of 3. At the indicated times p.i., cells were fixed and stained for nucleus (Hoechst), mitochondria (Tom20), vRNPs (using NP as proxy) and cell membrane (WGA). Images were acquired using Leica SP5. Each confocal image was converted from polar to Cartesian system using the automatic tool. (B) Representative images at 8h post-infection. Bar=10μm. (C) The percentage of perinuclear mitochondria (within 2μm of the nucleus) was plotted over the course of infection. (D) The percentage of cell peripheral mitochondria (4μm from the cell nucleus) at 8h p.i. was also plotted. An average of 100 cells per time-point was processed to obtain the graphs in (B) and (C). Error bars represent the standard error of the mean (S.E.M.). Statistical analysis of data was performed using non-parametric Mann-Whitney test (***p<0.001; ns, non-significant).

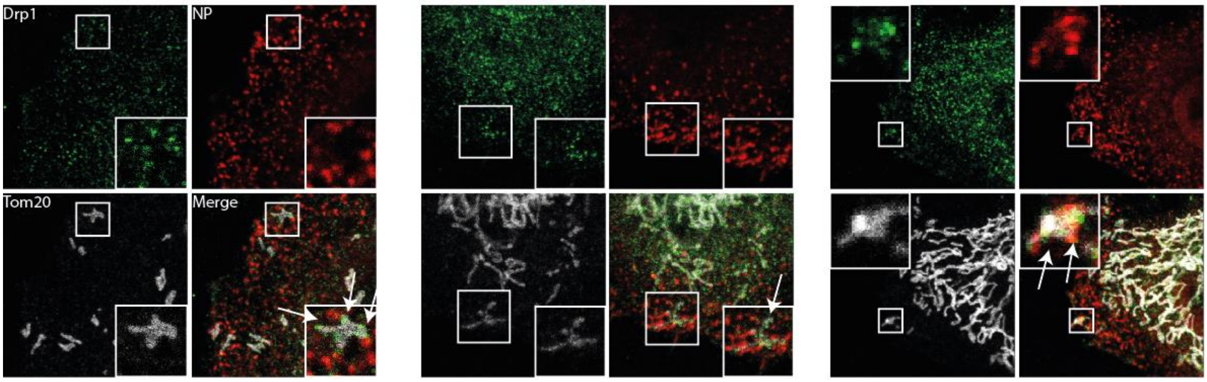

Figure 3- vRNPs associate with mitochondria.

HeLa cells were infected with PR8 at a MOI of 5. At 8 h p.i. cells were fixed and stained for vRNPs (using NP as proxy, red), mitochondria (Tom20, grey) and Drp1 (green). Images were acquired with Leica SP5 confocal microscope. Inlet show areas of vRNP/mitochondria/Drp1 aggregation in 3 independent cells. Arrows show areas of contact. Note that Drp1 may be establishing a bridge between mitochondria and vRNPs.

Additionally, the comparison of infected WT A549 cells with infected GFP-Rab11A A549 cells in Fig S1 raises some concerns regarding potential non-significant effects of Rab11A

overexpression, and a more thorough analysis would be valuable (more small vesicles? Are these structures behaving the same all over the cell?).

We have previously published a full characterization of the GFP-Rab11a WT cell line (in A549 background) [4]. In comparison to A549 cells, GFP-Rab11a cells assemble larger viral inclusions given that more Rab11a vesicles are accumulating in viral inclusions. This cell line has been key in correlative light and electron microscopy studies and immunogold labeling, allowing the clear identification of the viral inclusions in the cytoplasm of the infected cell [4]. In the current manuscript, we meant to show that expression of low levels of GFP-Rab11a do not interfere with the formation of DMVs.

Finally, what is the evidence and relevance of the structures labelled as vacuoles being a product of ER enlargement? Regardless of all these questions and the potential answers the authors could provide, how relevant is these data to the hypothesis being tested? It is not clear the rationale of these experiments.

The data on ER enlargement is not relevant for this manuscript and we have removed reference to it, although it will remain marked in the model.

6. There is a potential discrepancy in the observations made about Fig 1C (L 160-164), as the dmvs appear to label both small and big gold particles (and not "mostly"), if one observes not only the structures highlighted with an arrowhead: the surrounding dmvs have almost the same amount of both particles (quantification?). But once again, what is the relevance and novelty of this observation with the overall hypothesis (where's the ER? Haven't the authors already shown Rab11A and vRNPs co-localization in the past (and later in this paper)?). Also, the labeled smv is almost the same size as the dmvs in Fig 1C, so how does this observation fit Fig 1A results? This whole Figure 1 or the text explaining it needs to be revisited in the context of the rest of the manuscript.

IAV liquid inclusions or condensates are composed of aggregated vesicles similar to those observed for synaptic vesicles [5]. We published before that the vesicles accumulating are heterogenous in size and therefore it is expected to observe a wide range of SMV sizes. SMVs are highlighted as every light green structure in the image (Fig 2 of updated manuscript version). The “ER vacuole”, now designated “ER dilation” is dark green.

Tokuyasu immunogold is not a quantitative methodology, as many epitopes are damaged during the sample processing. We have chosen to include this in supplementary rather than in the main manuscript (S2B Fig in updated version).

7. Another potential conflict I have with the way the story is presented and arising from the results in Fig 1 is that authors claim that these vRNPs are liquid-liquid phase separated organelles but then they show that actually these structures are dmvs or are surrounded by a dmv. How is this possible then?

IAV liquid inclusions are a liquid organelle or condensate formed by the aggregation of recycling endocytic vesicles (Figure 1 below), similar to what happens in synaptic vesicles in neurons ready for deployment upon stimuli [5]. The accumulated vesicles share properties with *bona fide* liquid condensates. They are not delimited by a membrane (although they contain membranes inside), are dynamic, react to stimuli and fuse and divide. Both Rab11A and vRNPs co-localize in these condensates [4].

Our proposed model is that during infection, viral progeny RNA (vRNPs) binds to Rab11a-GTP on recycling endosomes, and recycling endosome vesicles, rather than being transported to the plasma membrane, accumulate close to ER exit sites. This means that the Rab11a recycling activity is impaired with infection. In our model, the liquid-like character results from a network of weakly interacting vRNPs that bridge multiple cognate vRNP-Rab11 units on flexible membranes [7]. This has important consequences on how IAV genomic complexes

are formed, that are composed of eight different segments, and constitutes a change in paradigm.

We will include this information in the manuscript to help understand our model.

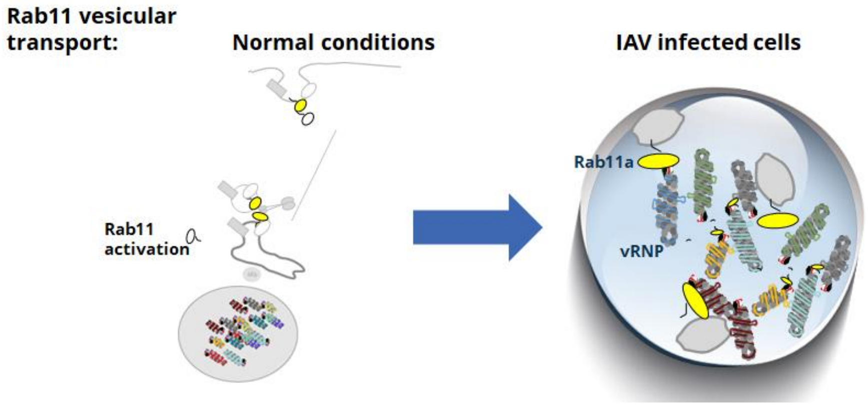

Figure 1. IAV inclusions have the properties of liquids.

In normal conditions, Rab11a vesicles are able to recycle cargo to the plasma membrane using the microtubule network. Upon IAV infection, vRNPs bind Rab11a (via PB2 viral protein) and as the cytoplasmic content of vRNPs increases [8,12–15], vRNPs outcompete Rab11a adaptors/molecular motors for Rab11a binding [8–10]. As a consequence, recycling is impaired during infection [10] and Rab11a vesicles bound to vRNPs are re-routed to ER exit sites to form viral inclusions that display the properties of liquid condensates [4,17].

8. L 199: using a WB for ATG9A would be more appropriate than RTqPCR to show the efficiency of knockdown, especially for transmembrane proteins like ATG9A.

We have included western blot quantifications whenever possible. For some proteins we could not obtain reliable antibody stainings, so we opted for RT-qPCR analysis.

9. In relation to Fig 2C and 2E-F, there is a noticeable increase in NP-positive structures in ATG9A KD cells which the authors do not address. This could suggest the possibility that autophagy clears some of these inclusions in the WT and also explain why ATG9A re-distributes upon infection (i.e., autophagy activation and recruitment to autophagosomes). Since ATG9A depletion doesn't affect Rab11A and NP colocalization and doesn't affect virion

assembly and packaging and the titers are marginally smaller in the KD versus the siNT conditions, ATG9A seems to have a downstream effect of vRNP assembly, which is clearly against the original hypothesis. The speculation about ATG9A influencing the affinity of viral inclusions to the microtubule network is not mechanistically addressed: **how is it that a scramblase affects cargo-microtubule dynamic?** If it's not through the scramblase activity, what other property or quality of ATG9A and ATG9A-vesicles could be responsible for this particular function? **Are other cargo-microtubule interactions being affected or this is specific for IAV viral inclusions?** These important questions are not fully addressed in the paper. On the contrary, authors decide to **speculate that "ATG9A controls the exit of viral inclusions from microtubules to the ER"** (L567-568) but this argument is also poorly supported and hard to understand. **How is the viral inclusion exiting a microtubule? How is that the viral inclusion goes to or enters the ER?** Even more, depletion of ATG9A could have completely undirect effects (but still related to autophagy deficiency?) leading to a viral assembly machinery and interactions that persevere in the absence of ATG9A, as supported by Figs. 4C and D, 5 and 6, making these phenotypes not as relevant.

We thank the reviewer for prompting us to consider new hypotheses that will be discussed in the relevant section of the manuscript. Indeed, our model is that ATG9A responds to some alteration in the infected cell that initiates a cascade of events, culminating in the accumulation of recycling endocytic vesicles close to the ER. We do not think that ATG9A directly modifies Rab11a recycling endosomes. In fact, in the context of ATG9A overexpression, ATG9A localizes between the ER and IAV inclusions. This raises the hypothesis that when the normal function of ATG9A is not happening at the ER, Rab11a vesicles do not accumulate at ERES, do not form optimal viral inclusions and circulate in microtubules. The concept that ATG9A is a modulator of liquid-liquid phase separation on or near ER in mammalian cells has also been recently proposed by another group [3]. ATG9A regulates phase separation and spatial organization of the autophagosome component FIP200 on the ER tubules [3]. This paper does not explore further the function of ATG9A. This has been explored in the discussion of our

manuscript. In this case, the function was linked to autophagy, but in our case, given that ULK1/2, ATG2A and TBC1D14 did not produce a similar phenotype, we think that the signal is different. In a collaborative work led by Petr Chlanda (Univ. Heidelberg), we have interesting data on how the DMVs are formed, but we can't disclose them at the moment. Given the extent of the manuscript, we think that the function of ATG9A is outside the scope of the present work.

We think that the observed effect is specific to IAV infection, as we observe accumulation of Rab11a (and b) vesicles close to ER exit sites, but not of vesicles marked by Rab5, Rab7, Rab9, Rab25, Rab4 (and many other Rabs). Depleting them does not affect IAV production. In agreement, a recent study showed that Rab11a positive vesicles recruited less dynein in infection when compared to uninfected cells [16].

This reviewer raises the point that ATG9A depletion doesn't affect Rab11a and NP colocalization and doesn't affect virion assembly and packaging. We respectfully disagree with the last part of the sentence. Virions that are released from the cell contain equal numbers of fully infectious viral particles, but that does not mean that the efficiency of IAV genome assembly happening inside the cell is that same. If our model is correct, ATG9A depleted cells would contain more vRNPs in the cytosol than control cells. This reviewer observed that, in former Fig 2C and 2E-F, there is a noticeable increase in NP-positive structures in ATG9A KD cells, and this supports our hypothesis. We have quantified the amount of vRNPs in cells depleted or not of ATG9 to support our hypothesis and found there is accumulation of vRNPs in the cell cytosol upon ATG9A depletion (Fig 7A-B new manuscript version).

We propose to clearly address all these different hypotheses in the discussion, pinpointing which data is still missing to formally validate our model.

References

[1] Zhao YG, Zhang H. Phase Separation in Membrane Biology: The Interplay between Membrane-Bound Organelles and Membraneless Condensates. Dev Cell 2020;55:30–44. https://doi.org/10.1016/j.devcel.2020.06.033.

[2] Lee JE, Cathey PI, Wu H, Parker R, Voeltz GK. Endoplasmic reticulum contact sites regulate the dynamics of membraneless organelles. Science 2020;367:eaay7108. https://doi.org/10.1126/science.aay7108.

[3] Zheng Q, Chen Y, Chen D, Zhao H, Feng Y, Meng Q, et al. Calcium transients on the ER surface trigger liquid-liquid phase separation of FIP200 to specify autophagosome initiation sites. Cell 2022. https://doi.org/10.1016/j.cell.2022.09.001.

[4] Alenquer M, Vale-Costa S, Etibor TA, Ferreira F, Sousa AL, Amorim MJ. Influenza A virus ribonucleoproteins form liquid organelles at endoplasmic reticulum exit sites. Nat Commun 2019;10:1629. https://doi.org/10.1038/s41467-019-09549-4.

[5] Milovanovic D, Wu Y, Bian X, De Camilli P. A liquid phase of synapsin and lipid vesicles. Science 2018;361:604–7. https://doi.org/10.1126/science.aat5671.

[6] Etibor TA, Yamauchi Y, Amorim MJ. Liquid Biomolecular Condensates and Viral Lifecycles: Review and Perspectives. Viruses 2021;13:366. https://doi.org/10.3390/v13030366.

[7] Etibor TA, Vale-Costa S, Sridharan S, Brás D, Becher I, Mello VH, et al. Defining basic rules for hardening influenza A virus liquid condensates. ELife 2023;12:e85182. https://doi.org/10.7554/eLife.85182.

[8] Amorim MJ, Bruce EA, Read EKC, Foeglein Á, Mahen R, Stuart AD, et al. A Rab11- and Microtubule-Dependent Mechanism for Cytoplasmic Transport of Influenza A Virus Viral RNA. J Virol 2011;85:4143–56. https://doi.org/10.1128/JVI.02606-10.

[9] Veler Hana, Fan Haitian, Keown Jeremy R., Sharps Jane, Fournier Marjorie, Grimes Jonathan M., et al. The C-Terminal Domains of the PB2 Subunit of the Influenza A Virus RNA Polymerase Directly Interact with Cellular GTPase Rab11a. J Virol 2022;96:e01979-21. https://doi.org/10.1128/jvi.01979-21.

[10] Vale-Costa S, Alenquer M, Sousa AL, Kellen B, Ramalho J, Tranfield EM, et al. Influenza A virus ribonucleoproteins modulate host recycling by competing with Rab11 effectors. J Cell Sci 2016:jcs.188409. https://doi.org/10.1242/jcs.188409.

[11] Kawaguchi A, Hirohama M, Harada Y, Osari S, Nagata K. Influenza Virus Induces Cholesterol-Enriched Endocytic Recycling Compartments for Budozone Formation via Cell Cycle-Independent Centrosome Maturation. PLoS Pathog 2015;11:e1005284.

[12] Avilov SV, Moisy D, Munier S, Schraidt O, Naffakh N, Cusack S. Replication-Competent Influenza A Virus That Encodes a Split-Green Fluorescent Protein-Tagged PB2

Polymerase Subunit Allows Live-Cell Imaging of the Virus Life Cycle. J Virol 2012;86:1433–48.

[13] Chou Y-Y, Heaton NS, Gao Q, Palese P, Singer R, Lionnet T. Colocalization of Different Influenza Viral RNA Segments in the Cytoplasm before Viral Budding as Shown by Single-molecule Sensitivity FISH Analysis. PLoS Pathog 2013;9:e1003358.

[14] Eisfeld AJ, Kawakami E, Watanabe T, Neumann G, Kawaoka Y. RAB11A Is Essential for Transport of the Influenza Virus Genome to the Plasma Membrane. J Virol 2011;85:6117–26.

[15] Lakdawala SS, Wu Y, Wawrzusin P, Kabat J, Broadbent AJ, Lamirande EW, et al. Influenza a virus assembly intermediates fuse in the cytoplasm. PLoS Pathog 2014;10:e1003971.

[16] Bhagwat AR, Le Sage V, Nturibi E, Kulej K, Jones J, Guo M, et al. Quantitative live cell imaging reveals influenza virus manipulation of Rab11A transport through reduced dynein association. Nat Commun 2020;11:23.

[17] Etibor TA, Vale-Costa S, Sridharan S, Brás D, Becher I, Mello VH, et al. Rules for hardening influenza A virus liquid condensates. BioRxiv 2022:2022.08.03.502602. https://doi.org/10.1101/2022.08.03.502602.

[18] Beale R, Wise H, Stuart A, Ravenhill BJ, Digard P, Randow F. A LC3-Interacting Motif in the Influenza A Virus M2 Protein Is Required to Subvert Autophagy and Maintain Virion Stability. Cell Host Microbe 2014;15:239–47. https://doi.org/10.1016/j.chom.2014.01.006.

[19] Snead WT, Gladfelter AS. The Control Centers of Biomolecular Phase Separation: How Membrane Surfaces, PTMs, and Active Processes Regulate Condensation. Mol Cell 2019;76:295–305. https://doi.org/10.1016/j.molcel.2019.09.016.

[20] de Castro Martin IF, Fournier G, Sachse M, Pizarro-Cerda J, Risco C, Naffakh N. Influenza virus genome reaches the plasma membrane via a modified endoplasmic reticulum and Rab11-dependent vesicles. Nat Commun 2017;8:1396. https://doi.org/10.1038/s41467-017-01557-6.
