# Supplementary material for: ATG9A regulates the dissociation of recycling endosomes from microtubules to form liquid influenza A virus inclusions
Source: PLoS Biol. 2023 Nov 20;21(11):e3002290. doi: 10.1371/journal.pbio.3002290 (PMC10695400; doi:10.1371/journal.pbio.3002290)
Supplement: Attachment — Submitted filename: Response to Reviewers.pdf [file pbio.3002290.s030.pdf]

Response to Reviewers - PBIOLOGY-D-23-01900R1

We thank the reviewers for the thoughtful and thorough assessment of our manuscript. We are pleased that the revisions we performed aligned with the expectations of the reviewers, and it is with great pleasure that we have received positive feedback for publication in PLoS Biology.

We understand that there are still some concerns that the reviewers would like to see addressed, to which we provide an answer under the respective question written in blue.

We hope that these clarifications and modifications performed in the second revision and/or our answers to the queries will be well received for final acceptance in PLoS Biology and but we remain at your disposal to address any further inquiries or concerns.

Please also address the following formatting and journal policy requests:

1. We suggests a change in the title: "ATG9A regulates the dissociation of recycling endosomes from microtubules to form influenza A virus liquid condensates" or "ATG9A regulates the dissociation of recycling endosomes from microtubules to form liquid influenza A virus inclusions".

We have changed the title as suggested in the latter option.

2. Please provide a blurb which (if accepted) will be included in our weekly and monthly Electronic Table of Contents, sent out to readers of PLOS Biology, and may be used to promote your article in social media. The blurb should be about 30-40 words long and is subject to editorial changes. It should, without exaggeration, entice people to read your manuscript. It should not be redundant with the title and should not contain acronyms or abbreviations.

Influenza A virus infection can trigger host recycling endosome condensation juxtaposed the endoplasmic reticulum. We demonstrate a regulated process involving ATG9A, facilitating the detachment of recycling endosomes from microtubules close the endoplasmic reticulum, showcasing a novel role for ATG9A beyond autophagy.

REVIEWS:

Reviewer #1:

We thank the reviewer for providing feedback to our manuscript and for recognizing the efforts we made to address the reviewers' concerns in this revised version of our work. It is a fact that one unresolved aspect in this report pertains to the molecular mechanism by which ATG9 facilitates the removal of IAV viral inclusions from microtubules. While we acknowledge this gap, we have explicitly stated it in the manuscript and presented potential directions for future investigation. We appreciate the reviewer's understanding and agreement that this approach aligns with the maturity required for publication.

In connection to the specified points still raised, kindly locate the respective answers beneath each one in blue.

An important step in Influenza A virus (IAV) biogenesis is the coalescing of new viral genome segments and viral NP protein in condensates in the cytoplasm. These condensates have previously been shown to form at ER exit sites, move along microtubules, and contain Rab11 - a small GTPase that is normally associated with recycling endosomes.

In this paper, Vale-Costa report that the autophagy protein ATG9A plays a role in condensate dynamics. ATG9A is a lipid scramblase that is thought to facilitate the growth of the phagophore membrane in autophagy, in addition to having some seemingly non-autophagic functions. The effect of ATG9A on IAV replication is not dramatic (in terms of its effect on virus release), but the cell biology is still interesting.

The core finding in this manuscript, in my opinion, is the altered appearance and dynamics of IAV condensates upon siRNA depletion of ATG9A. The condensates become drawn out, and the authors use beautiful live-cell imaging to show that this is due to increased association with microtubules.

I shall be frank and say that I reviewed a previous version of this manuscript for PLOS Biology, and even though this is formally considered a new submission, the authors have revised the manuscript according to the reviewers suggestions.

In my opinion, the new manuscript is substantially better in many ways. It is easier to follow the logics of the paper, and some crucial additional experiments tie the paper together better than before. Of my major criticisms to the previous version of the manuscript (I assume they will be published as part of the rebuttal letter), most have been dealt with satisfactorily. The only major point I made that the authors didn't address is the following: "The paper lacks mechanistic detail as to how ATG9 might have its effect on IAV condensates. The fluorescence microscopy is largely well made, but the kind of proximity of fluorescence signals shown in Fig. 3F is not a proof of anything. The authors must provide more insights into how ATG9 is involved. One example of such insights would be convincing proof of interaction between ATG9 and some viral proteins." Since that concern was not addressed, the new manuscript leaves one elephant in the room untouched: how (in terms of biochemical interactions) can ATG9A, a lipid scramblase, regulate the association of vRNP condensates to microtubules and ER? However, in the new manuscript the authors clearly discuss that this is not resolved, they put it in the context of similar phase separation phenomena, and they put forward some ideas related to regions of ATG9A. Since my other major concerns were addressed, the paper has reached a maturity that, in my opinion, warrants publication in PLOS Biology.

I still have some remaining smaller comments that I think the authors can sort out between themselves and the editor without my re-re-review:

1. Simply as a comment to editor and authors: even though this is formally a new submission, it would really have helped the reviewers to get a manuscript where changes are highlighted. It takes several hours longer for a reviewer to carefully re-assess the manuscript when this is not done.

We understand that presenting the changes in a highlighted format would facilitate the reviewers in recognizing the modifications. Unfortunately, we were unaware that the PLoS submission system allowed us to include a 'track-changes' version of the previous version of the manuscript in a new submission.

The prior version of the manuscript was rejected by PLoS Biology, leaving however a door opened for submitting the work from start if we addressed reviewers' comments. Acting on the editor's advice, we engaged in a constructive dialogue to strategize our approach for resolving the reviewers' concerns. Our proposed plan was considered suitable, motivating us to proceed with experiments and new submission. We included the previous references to aid in easy tracking of reviewers and their comments as indicated in the guidelines.

2. "However, we find that key initial players in autophagy (ULK1/2, TBC1D14 or ATG2A) did not give rise to a similar phenotype in IAV infected cells, suggesting that this function of ATG9A is novel" I think this and some other similar statements are misleading the reader given that ULK2 had a larger effect on virion production than ATG9A. What they authors say is not directly incorrect, but it might be better to rephrase this to make clear that ULK2 has a role, but probably another one.

The sentence has been changed to accommodate the comment of the reviewer by changing it to: "However, we find that key initial players in autophagy (ULK1/2, TBC1D14 or ATG2A) did not regulate ER-microtubule trafficking, even though ULK2 affected virion production, suggesting that this function of ATG9A is novel".

3. Comment to the last paragraph on page 20: The authors don't show any quantitation here. I assume that it would not work since it looks like the overexpressed ATG9A-EGFP is simply all over the cytoplasm. I think the data can be kept but the limitation of this experiment in terms of possibly different a priori distribution of ATG9 should be thoroughly mentioned.

The sentence has been changed to accommodate the limitations of overexpressing ATG9. It now reads:

"Even though we acknowledge the limitations of overexpression experiments, in the sense that may change the cell and lead to an alteration of the cellular distribution of ATG9A, we could confirm that GFP-ATG9A strongly localized at the Golgi in mock-infected cells, and that this co-localization is lost with infection (Fig 4D), as observed for endogenous ATG9 (Fig 4A). This observation reinforces the data on GFP-ATG9A establishing multiple contacts with viral inclusions identified by NP and Rab11a (Fig 4E). This pattern resembles the one we previously described using ERES markers (Sec16 and Sec31) (Alenquer et al., 2019). Moreover, ATG9A puncta were found in the vicinity of viral inclusions and the ER (inset in Fig 4F)."

4. Comment about the subheading "ATG9A impacts viral inclusion formation without affecting the recycling endosome". This description is imprecise and clearly overstating what the authors have shown. It should be changed to something more correct and moderate. The tile of Fig. 5 is also an overstatement since the authors have hardly experimentally verified that "Rab11a-recycling", in all the meanings of that term, is unaffected.

As suggested by the reviewer, we altered the subheading to "ATG9A impacts viral inclusion formation without affecting the binding of vRNPs to the recycling endosome" and the title of Fig. 5 to ATG9A impacts viral inclusions independently of Rab11a-vRNP interaction.

5. "This defect, however is unlikely to be related to the recycling endosome as ATG9A depletion did not interfere with the association of vRNPs to Rab11a vesicles." See comment above. This interpretation is too broad.

We changed the sentence to be precise to “This defect, however, is unlikely to be related to the association of vRNPs to Rab11a vesicles as ATG9A depletion did not interfere with the colocalization and spatially synchronized dynamic movement of vRNPs-Rab11”.

6. "We observed that in siNT-treated and infected cells, viral inclusions became larger with little motility upon nocodazole treatment" The authors should either support this statement with quantitation, or change it.

In Fig 6H we show mean square displacement of viral inclusions treated with and without nocodozale and therefore the motility upon nocodazole treatment is quantified. However, we assigned Fig 6H and its experimental design Fig 6G to the text, as it was missing. As we provided quantitation of area of nocodazole treated viral inclusions in two previous manuscripts, we have now included their citation in the text, and changed the text to say that we confirmed our previous results. We hope that these alterations will be considered suitable.

The text has been modified as follows: First, we confirmed our previous results in which in siNT-treated and infected cells, viral inclusions became larger with little motility upon nocodazole treatment (Fig 6E-H)(Amorim et al., 2011; Etibor et al., 2023).

7. Comment on the paragraph "ATG9A impacts efficiency of viral genome assembly but not genome packaging into virions ": I think it would help the readers to clearly define what the authors mean by "genome assembly" and "genome packaging". These terms are widely used in virology and usually have a different meaning than here. They are often even synonymous.

We have explained in the text what we mean by genome assembly and genome packaging as follows: Given that viral inclusions are seen as the putative sites where the complex of the IAV segmented genome, comprising eight different vRNPs, is formed (viral genome assembly), we hypothesized that the arrest of viral inclusions at microtubules caused by ATG9A depletion would affect late steps of viral infection. The late stages include genome assembly, viral surface protein levels and the inclusion of the assembled genome (genome packaging) into budding virions.

8. Comment on Fig. 8: The figure and its legend would benefit from clarifications. It should be described explicitly what the two versions of the cell represent, and also what the arrow from one version to the other represents.

We thank the reviewer for asking for further clarifications relative to our model. We have now described what the two images mean and included numbers to explain each of the steps we propose, as follows:

Fig 8. Proposed model for ATG9A role in the establishment of liquid IAV inclusions.

We currently view liquid viral inclusions, composed of Rab11a endosomes and viral ribonucleoproteins (vRNPs), as sites dedicated to the assembly of the influenza A virus genome (Alenquer et al., 2019; Amorim, 2019; Vale-Costa et al., 2016). We have previously shown that liquid viral inclusions develop in close contact with the endoplasmic reticulum exit sites (ERES) (Alenquer et al., 2019). Here we describe the initial events on the left panel that may lead to the formation of liquid viral inclusions on the right to facilitate the formation of IAV genomic complex. In this study, we demonstrate that IAV

infection reduces the Rab11a-regulated recycling capacity of the host cell (step 1). This effect is likely a consequence of vRNP binding to Rab11a endosomes, which are then re-routed to the ERES to form viral inclusions. Such trafficking of Rab11a endosomes carrying the vRNPs to the ER is regulated by the host factor ATG9A. We identified that ATG9A is mobilized from the Golgi during IAV infection (step 2) and leads to the removal of Rab11a-vRNP complexes from microtubules when at the ER (step 3). It is thus possible that ATG9A moves to the ER to promote the linkage of viral inclusions to microtubules. In this location, vRNPs-Rab11a units may establish multiple and dynamic contacts forming liquid percolation-driven condensates. We also show (although with overexpression experiments) that ATG9A engages in multiple contacts with viral inclusions (step 4). We propose that the liquid properties of viral inclusions favour the formation of the 8-segmented IAV genome that is transported to the plasma membrane (step 5).

9. "In sum, our results demonstrate that the recycling pathway is impaired during IAV infection. Moreover, enlarged cytosolic Rab11a puncta (corresponding to the liquid viral inclusions) are detected near the ER only in infected cells expressing an active Rab11a, which agrees with our previous results [4,6,7]." The Rab11a-related statement now appears before these results (new Fig. 1D-E) are discussed in the text. That should be changed.

The results of cytosolic puncta or viral inclusions close to the ER are also presented in S1B Fig, which precedes this sentence. Therefore, we think that the sentence is correctly placed.

10. "How assembled genomes reach the budding sites at the plasma membrane is not yet known, but such a question is outside the scope of this study (Fig 1E, step 4)" Should it not be Fig. 1A?

Yes, it should be Fig 1A and it has now been fixed. Thank you.

Reviewer #2:

We thank this reviewer for providing insightful feedback to our manuscript and for pointing additional concerns that will lead to a clearer and more concise message. All points raised have been addressed and answered under each point raised.

We hope that they are aligned with your expectations regarding the manuscript.

The increased data from the silencing screen indicates specific effects of ATG9A rather than on autophagy. Overall, the text is easier to follow but some paragraphs are still difficult to understand. For example (Fig 1C, 1C) a transferrin recycling assay is used to see if IAV impairs Rab-11a regulated recycling. The conclusion below states that ''recycling occurs primarily independently of Rab11a''. So this makes it difficult to see how the assay is testing the effects of IAV on Rab11a.

''This indicates that transferrin recycling occurs primarily independently of Rab11a, through a compensatory mechanism likely mediated by other Rabs operating in the recycling pathway (Rab4, Rab10) [15] in uninfected cells but that in infection by itself reduces recycling of transferrin and dependency for Rab11a.''

We totally understand your point, and thanks for raising this issue. We have now clarified our text to say that it is the combined visual inspection of Rab11 redistribution from mock to infected cells together with the reduction in transferrin recycling in these cells expressing WT and more pronounced in DN Rab11 that allows determining that Rab11a recycling is impaired in infection.

We used transferrin recycling, as many manuscripts have provided evidence on the role of Rab11 in transferrin recycling. Green in 1997 found that Rab11 is associated with transferrin-containing recycling compartments in non-polarized cells. Schlierf 2000 demonstrated that Rab11b is essential for recycling transferrin to the plasma membrane. Ullrich 1996 showed that Rab11 regulates recycling through the pericentriolar recycling endosome. Takahashi 2012 revealed that Rab11 is involved in exocytosis of recycling vesicles at the plasma membrane. These findings highlight the importance of Rab11 in the recycling of transferrin and its role in regulating membrane trafficking (Green et al., 1997; Schlierf et al., 2000; Takahashi et al., 2012; Ullrich et al., 1996).

However, we were intrigued by our results. We think that the low dependency of transferrin recycling on Rab11a translates the complexity of cellular recycling, that involves Rab4, Rab10 (Mohrmann et al., 2002) and Rab11 families operating via many redundant pathways with our cells having an active Rab11b. In uninfected cells, we see a 12.5% reduction in transferrin recycling in Rab11 DN relative to Rab11 WT cells, and hence, we reach the conclusion that transferrin recycling in our system occurs primarily independently of Rab11a. In infection, we find that transferrin recycling is reduced to half in Rab11 WT and has an additional 25% decrease in Rab11 DN. Hence, in infection, transferrin recycling suffers a decrease of 75% in DN cells. This indicates a modest increase in dependence of transferrin recycling in Rab11a. vRNPs do not use Rab4 or Rab10. We know this because we (and others) have tested it and Rab4 or Rab10 do not affect viral titres (unpublished data). The dramatic effect on the redistribution of Rab11a that we and others have reported indicates that Rab11a recycling is impaired.

The rebuttal is supplied with diagrams describing interaction of vRNA with Rab11a and formation of viral inclusions, but the manuscript focusses on the role played by ER exit sites and ATG9A in tethering inclusions to microtubules. It is not clear why ER exit sites, ATG9A and microtubules do not feature in the diagram supplied for referees. Fig 8 in the manuscript provides a more detailed diagram. Addition of numbers to the arrows would have made this easier to navigate.

This is a very long paper and would benefit from editing to cut out repetition between introduction and discussion. In this respect the Rab11a data, which takes up considerable space in terms of figures and text seems to confirm what has been published by this group and others. The data show that Rab11a and Atg9A work independently of each other so the authors might consider reducing the emphasis on Rab11a data that is in agreement with published work, and focus on ATtg9A.

We have cut the introduction and the discussion as much as possible and still maintain the clarity of the message we want to convey. Despite the independence of ATG9A from Rab11a, we find that the differences found by removing ATG9 need to be put into context with Rab11a trafficking as in our model, without Rab11a binding, vRNPs would not reach the endoplasmic reticulum (ER). We were still able to cut considerably the discussion.

Referee response to rebuttal.

The specific comments have been addressed.

1. ''The first experiments describe the use of high pressure freezing and EM tomography to provide 3D reconstructions of ER:LO interface. New double membraned vesicles, single membrane vesicles and enlarged ER lumen are seen close to the ER.''

It is not clear how this relates to LOs that do not have a limiting membrane.

This has been clarified in the text.

Thank you for agreeing with the modifications we made concerning this issue.

2. ''The text ln163 says 'It is noteworthy that numerous vRNPs detected inside viral inclusions were not attached to any membrane (Fig 1C). '' These are not indicated in the figure. At this point the data suggest that Rab11 is a marker for membrane compartments, rather than LO.

The use of Rab11 as a marker is now explained much better.

Thank you.

3. ''A line scan follows fusion of LO with the ER in infected cells but does not follow interaction of Rab11 with ER in uninfected cells. The videos are difficult to interpret. A line scan for interaction in mock infected cells should be provided to support the main conclusions that ER contacts are increased for Rab11 after infection.

New line scans have been introduced. In Fig 1E.

Thank you for acknowledging our revised approach regarding this matter.

4. ''Silencing of ATG9A produces a small drop in viral titre and converts LO into tubular networks and appears to reduce association of LO with ER. Here the virus is tracked with antibodies to NP. Some explanation for why the experiments track NP, rather than Rab11, is needed.

The reduced signal to noise ratio generated by NP staining provides a logical explanation.

Thank you for agreeing with the modifications we made concerning this issue.

5. ''LO contacts are reduced following silencing of ATG9A. '' The quantification is difficult to follow because arrows in figure 2E and 2F indicate different events making comparison difficult. Is the conclusion based on a visual interpretation where rounded LO are easier to image than tubular LO, or it is easier to surround a round LO rather than a tube, without making contacts?

An explanation is provided and shapes ''roundness'' have been re calculated.

Thank you for endorsing our revised approach regarding this particular concern.

6. ''The paper concludes '' that ATG9A is critical for proper establishment of IAV inclusions at the ER but is unlikely to be mobilized from the recycling endosome nor does it influence the association of vRNPs to Rab11a vesicles.''' This is difficult to follow because the images do not look at the ER or the effect of loss of recycling endosomes following expression of DN Rab11.

The roles of Rab11a and ATG9A have been explained more clearly emphasising that the results show that ATG9A and Rab11a have independent functions.

Thank you for approving our new explanation.

7. ''Again the text refers to Rab11 vesicles which most cell biologists would think are recycling endosomes rather than liquid organelles. At other times they are called viral inclusions, but are these liquid organelles?''

These changes in terminology have been clarified.

Thank you approving our clarification.

References for the response to reviewers:

Alenquer, M., Vale-Costa, S., Etibor, T.A., Ferreira, F., Sousa, A.L., Amorim, M.J., 2019. Influenza A virus ribonucleoproteins form liquid organelles at endoplasmic reticulum exit sites. Nat. Commun. 10, 1629. https://doi.org/10.1038/s41467-019-09549-4

Amorim, M.J., 2019. A Comprehensive Review on the Interaction Between the Host GTPase Rab11 and Influenza A Virus. Front. Cell Dev. Biol. 6, 176. https://doi.org/10.3389/fcell.2018.00176

Amorim, M.J., Bruce, E.A., Read, E.K.C., Foeglein, Á., Mahen, R., Stuart, A.D., Digard, P., 2011. A Rab11- and Microtubule-Dependent Mechanism for Cytoplasmic Transport of Influenza A Virus Viral RNA. J. Virol. 85, 4143–4156. https://doi.org/10.1128/JVI.02606-10

Etibor, T.A., Vale-Costa, S., Sridharan, S., Brás, D., Becher, I., Mello, V.H., Ferreira, F., Alenquer, M., Savitski, M.M., Amorim, M.-J., 2023. Defining basic rules for hardening influenza A virus liquid condensates. eLife 12, e85182. https://doi.org/10.7554/eLife.85182

Green, E.G., Ramm, E., Riley, N.M., Spiro, D.J., Goldenring, J.R., Wessling-Resnick, M., 1997. Rab11 Is Associated with Transferrin-Containing Recycling Compartments in K562 Cells. Biochem. Biophys. Res. Commun. 239, 612–616. https://doi.org/10.1006/bbrc.1997.7520

Mohrmann, K., Gerez, L., Oorschot, V., Klumperman, J., Van Der Sluijs, P., 2002. rab4 Function in Membrane Recycling from Early Endosomes Depends on a Membrane to Cytoplasm Cycle. J. Biol. Chem. 277, 32029–32035. https://doi.org/10.1074/jbc.M203064200

Schlierf, B., Fey, G.H., Hauber, J., Hocke, G.M., Rosorius, O., 2000. Rab11b Is Essential for Recycling of Transferrin to the Plasma Membrane. Exp. Cell Res. 259, 257–265. https://doi.org/10.1006/excr.2000.4947

Takahashi, S., Kubo, K., Waguri, S., Yabashi, A., Shin, H.-W., Katoh, Y., Nakayama, K., 2012. Rab11 regulates exocytosis of recycling vesicles at the plasma membrane. J. Cell Sci. jcs.102913. https://doi.org/10.1242/jcs.102913

Ullrich, O., Reinsch, S., Urbé, S., Zerial, M., Parton, R.G., 1996. Rab11 regulates recycling through the pericentriolar recycling endosome. J. Cell Biol. 135, 913–924. https://doi.org/10.1083/jcb.135.4.913

Vale-Costa, S., Alenquer, M., Sousa, A.L., Kellen, B., Ramalho, J., Tranfield, E.M., Amorim, M.J., 2016. Influenza A virus ribonucleoproteins modulate host recycling by competing with Rab11 effectors. J. Cell Sci. 129, 1697–1710. https://doi.org/10.1242/jcs.188409
